# Mutational pressure by host APOBEC3s more strongly affects genes expressed early in the lytic phase of herpes simplex virus-1 (HSV-1) and human polyomavirus (HPyV) infection

**DOI:** 10.1371/journal.ppat.1009560

**Published:** 2021-04-30

**Authors:** Maxwell Shapiro, Laurie T. Krug, Thomas MacCarthy

**Affiliations:** 1 Department of Applied Mathematics and Statistics, Stony Brook University, Stony Brook, New York, United States of America; 2 HIV and AIDS Malignancy Branch, Center for Cancer Research, National Cancer Institute, Bethesda, Maryland, United States of America; 3 Laufer Center for Physical and Quantitative Biology, Stony Brook University, Stony Brook, New York, United States of America; University of Minnesota, UNITED STATES

## Abstract

Herpes-Simplex Virus 1 (HSV-1) infects most humans when they are young, sometimes with fatal consequences. Gene expression occurs in a temporal order upon lytic HSV-1 infection: immediate early (IE) genes are expressed, then early (E) genes, followed by late (L) genes. During this infection cycle, the HSV-1 genome has the potential for exposure to APOBEC3 (A3) proteins, a family of cytidine deaminases that cause C>U mutations on single-stranded DNA (ssDNA), often resulting in a C>T transition. We developed a computational model for the mutational pressure of A3 on the lytic cycle of HSV-1 to determine which viral kinetic gene class is most vulnerable to A3 mutations. Using *in silico* stochastic methods, we simulated the infectious cycle under varying intensities of A3 mutational pressure. We found that the IE and E genes are more vulnerable to A3 than L genes. We validated this model by analyzing the A3 evolutionary footprints in 25 HSV-1 isolates. We find that IE and E genes have evolved to underrepresent A3 hotspot motifs more so than L genes, consistent with greater selection pressure on IE and E genes. We extend this model to two-step infections, such as those of polyomavirus, and find that the same pattern holds for over 25 human Polyomavirus (HPyVs) genomes. Genes expressed earlier during infection are more vulnerable to mutations than those expressed later.

## Author summary

The APOBEC family of cytidine deaminases are a component of innate immunity that can bind to viral DNA and cause mutations. We developed a computational model of the lytic life cycle of herpes simplex virus 1 (HSV-1), a large double-stranded DNA virus, to evaluate the potential effects of APOBEC on the virus and its evolution. The model considered three types of viral genes: Immediate Early (IE), Early (E) and Late (L). We found that genes expressed earliest in the lytic life cycle, E and particularly IE, are the most vulnerable to APOBEC-mediated mutations, because even low levels of mutation were sufficient to disrupt the infection process. We were able to validate the predictions of the computational model by analyzing published HSV-1 genomes, in particular by using software tools that quantify the extent to which each genome has evolved to reduce the number of DNA motifs (also known as “mutational hotspots”) that are preferred by APOBEC for mutation. Our analysis suggested that the IE and E genes have evolved to avoid APOBEC3 targeting. We extended our modeling and genomic analysis to human Polyomaviruses, which are smaller double-stranded DNA viruses with a simpler life cycle than HSV-1 and found similar results. These studies highlight the vulnerability to APOBEC of genes expressed early during viral infection and may suggest these genes as therapeutic targets.

## Introduction

Herpes Simplex Virus-1 (HSV-1), also known as human-herpesvirus 1, is a double-stranded DNA (dsDNA) virus, that undergoes productive lytic replication in epithelial cells and establishes latency in neuronal cells. HSV-1 infection may lead to minor lesions such as cold sores, or more serious conditions such as herpes encephalitis [1,2]. Reactivation from latency is associated with some cases of Bell’s Palsy [3]. Human Polyomaviruses (HPyV) are dsDNA viruses with a much smaller genome than HSV-1. Currently, there are four human polyomaviruses that have a clear association with diseases such as kidney and urinary tract disease in renal and bone marrow transplant patients, progressive multifocal leukoencephalopathy (PML), Merkel cell carcinoma, and Trichodysplasia spinulosa [4]. The error rate of the viral DNA polymerases of these dsDNA virus families are low, but co-speciation in vertebrates and variation among clinical isolates indicates that mutations arise, and positive selection pressures are more prevalent in DNA viruses than in RNA viruses [5,6]. The host factors that shape virus evolution are not well-defined for the dsDNA viruses. Here we focus on host-mediated cytosine deamination as a driver of genome editing and virus evolution.

In HSV-1 lytic infection, gene expression proceeds in a temporal order: immediate early (IE), then early (E), followed by late genes (L) [7]. Upon contact with the cell, the envelope fuses with the cell membrane, allowing the capsid to enter the cell via endocytosis [8]. From there, the virion travels along the microtubule network to the nucleus, wherein it injects its genetic material into the nucleus. Once in the nucleus, the viral genome rapidly circularizes. The tegument protein VP-16 binds to host Oct-1 and HCF-1 to recruit other cellular enzymes to derepress the IE promoters and initiate transcription of IE genes [4]. VP16, in association with host proteins, initiates transcription of the five IE genes: ICP0, ICP4, ICP22, ICP27, and ICP47 [7]. In particular, ICP4 acts as a key transcriptional regulator of all gene classes to enable transcription of both E and L genes while also acting to inhibit its own expression [9,10]. There are at least 12 E genes that are primarily responsible for nucleic acid metabolism and viral DNA replication. At least 56 L genes encode for structural and virion-associated proteins, including the tegument protein VP16 [11].

Lytic infection of HPyV proceeds temporally, in two phases that are transcribed from two distinct genomic regions termed early and late. Large T and small T antigens are expressed from the early region, whereas the late region expresses the viral coat proteins VP1, VP2, and VP3 [12]. The late region of several polyomaviruses also encodes miRNAs that function to target early mRNAs for degradation and limit the expression levels of T antigens [13]. A third region of the genome has a regulatory role and is often referred to as the non-coding control region [12].

The APOBEC3 (A3) enzymes, of which there are seven in human (A,B,C,D,F,G,H), belong to the AID/APOBEC family of cytidine deaminases [14]. The A3s have multiple roles in innate immunity, including restricting transposable elements and viral restriction by deamination of ssDNA or through deamination-independent mechanisms, such as disruption of reverse-transcriptase activity [15,16]. The A3 substrates are primarily single-stranded DNA (ssDNA) and, as such, have the potential to impact both transcription and/or replication of viruses that replicated in the nucleus. In the context of herpesviruses, ectopic expression of host A3C proteins induced lethal mutations on the HSV-1 genome *in vitro* [17].

To evaluate potential A3 pressures on viral evolution we previously developed the Cytidine Deaminase Under-representation Reporter (CDUR) software package to analyze A3 hotspot under-representation in viral sequences [18]. CDUR also determines viral susceptibility to amino acid changes, defined as an over-representation of non-synonymous changes in the coding sequence if the existing hotspots were to mutate. We recently reported that there is a duality of under-representation and susceptibility to A3B hotspots in a significant proportion of genes of the gammaherpesviruses Epstein-Barr Virus (EBV) and Kaposi’s sarcoma-associated herpesvirus (KSHV). EBV and KSHV evolved to limit the number of targets for A3B, resulting in genomic sequences that not only had fewer hotspots (under-representation) but whose remaining hotspots would cause mostly non-synonymous mutations that result in amino acid changes, presumably due to these sequences providing an essential role to the virus lifecycle. Little association was found between the temporal stage at which a viral gene was expressed and A3B-related features such as hotspot under-representation and mutation susceptibility, with the exception of four KSHV latent genes that did exhibit this duality [19,20]. As a possible counter-defense, the Epstein-Barr Virus (EBV) protein BORF2 inhibits A3B-related mutations by relocating A3B outside of the nucleus [21]. The BORF2 homolog in HSV-1, ICP6, does not appear to affect replication, despite the fact that it also relocates A3A/B [22].

Stochastic computational models have been widely used for viral infection models due to the inherently low numbers of molecules involved, particularly during the early stages of infection [23–26]. In the case of A3, mutations and their effects are inherently stochastic due to the randomness of ssDNA accessibility during transcription and/or translation [27,28], the uncertainty of deamination events following DNA binding [29], and differences in the consequences of deamination, whether the mutations are synonymous or non-synonymous [30]. Interestingly, A3A and A3B may have evolved to increase stochasticity via high-frequency conformational changes that occlude the catalytic pocket [31].

Here we investigate A3A/B targeting of HSV-1 and HPyV, and the consequences on the evolution of the viruses using a combination of computational modeling and sequence analysis. We developed a stochastic computational model and apply this model to investigate the impact of A3A/B-mediated mutations at specific stages of the virus replicative cycle. A previously published model [32] utilized ordinary differential equations (ODEs) and mass-action kinetics to describe the lytic cycle of HSV-1. We improve upon this model by adding various regulatory effects between gene classes, adding a probabilistic layer that determines A3 mutations, as well as convert it to a stochastic computational model. We find that A3A/B-induced mutations affecting the early stages of the lytic life cycle, specifically IE and E genes, tend to be more lethal to the virus than mutations in L genes. We next analyzed the HSV-1 for A3A/B hotspot under-representation and vulnerability to amino acid changes [18]. Based on the hotspot TC, which is characteristic of A3A/B, we find that the IE and E genes show the strongest effects of evolutionary pressure, significantly more so than the L genes, highlighting the particular importance of at least one of these two A3s in HSV-1 genomic evolution. We also evaluated a model of HPyV and find that viral fitness is more negatively affected by A3A/B mutations in early genes than in late genes. As with HSV-1, HPyVs have a genomic under-representation of A3A/B hotspots that is biased towards early genes rather than late genes, strongly supporting evolution to avoid A3A/B targeting.

## Results

### Motivation for a stochastic model for herpes simplex virus-1

HSV-1 primarily infects epithelial cells, where nuclear A3A/B are expressed [33]. Single-stranded DNA (ssDNA) would likely be accessible to nuclear A3A/B proteins during transcription and replication. IE and E genes are expressed prior to initiation of viral replication, followed by L gene expression. HSV-1 utilizes a rolling circle form of DNA replication, whereby the viral DNA polymerase traverses the circularized genome to make concatemeric DNA, though complex non-linear branched DNA replication intermediates exist as well [34]. We created a computational model of the viral lytic life cycle to investigate how A3 might disrupt the infection process, and how A3 may affect the viral fitness landscape under different mutational pressures.

**Fig 1:**
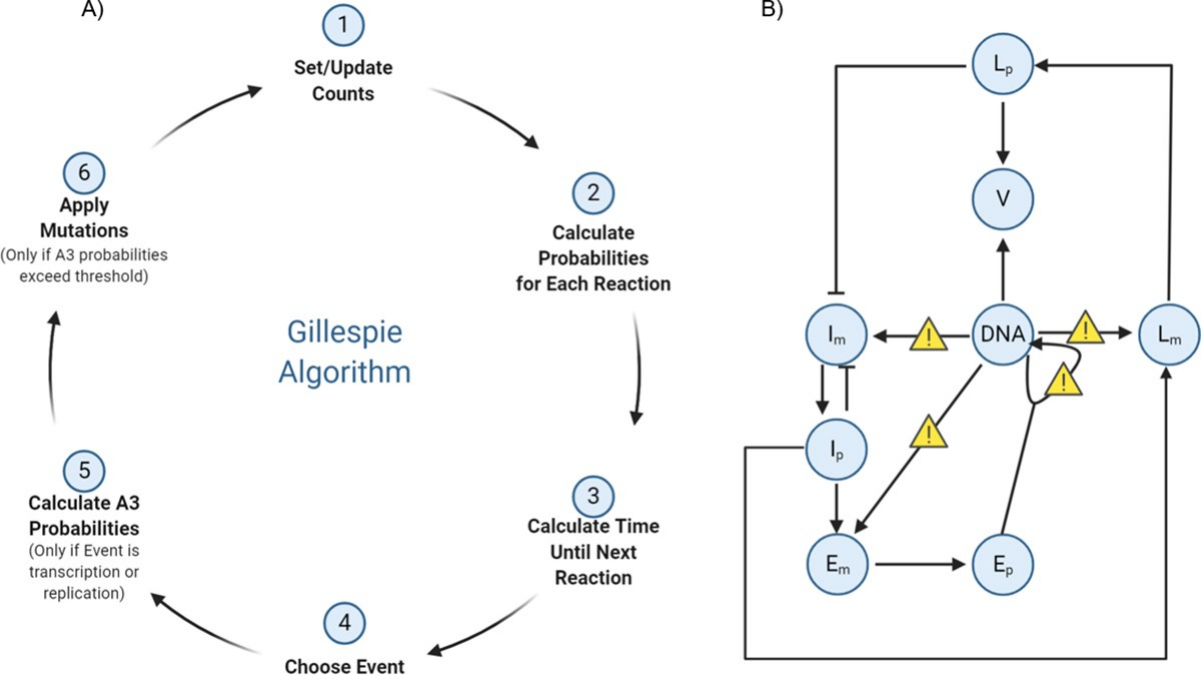
Gillespie Model implementation. The model is based on the ordinary differential equations (ODEs) in Eqs 1–8 (see Methods). A) Schematic diagram of Gillespie algorithm implementation. Starting with molecule counts, we calculate probabilities of a certain “reaction” event such as transcription of IE, E, or L genes; translation of mRNA; replication; or virion creation, among others. Next, we update time, and then randomly choose which event occurs from the previously calculated probabilities. Next, if a chosen event is a transcription or replication event susceptible to A3 mutation (here we consider transcription and replication), we set another probability of whether mutation from A3 occurs. Finally, we update the counts and repeat until a certain time threshold or a pre-defined number of cycles have passed. B) Components and interactions in the viral life cycle model. Where mutations may occur are denoted by yellow signs. Im, Ip denote IE gene mRNA and protein, respectively; similarly for Em,Ep,Lr, and Lp. V is virion.

The Gillespie algorithm [35], also known as the stochastic simulation algorithm, describes temporal changes in the numbers of molecules based on two criteria. First, the algorithm stochastically chooses which reaction will occur next from a given set of reactions, here based on regulatory interactions. Second, the algorithm determines the time lapse between reactions based on the total molecule count. Fig 1A illustrates the steps behind our implementation of the algorithm for the HSV-1 lytic cycle. At transcriptional and replicative steps, our algorithm probabilistically generates an A3 mutation deleterious to the virus. The regulatory interactions of the three genes (IE, E, L) in the model are described in Fig 1B. The model begins at the IE transcriptional step. IE proteins appear 2–4 hpi, while E proteins appear 5–7 hpi with viral DNA replication beginning shortly thereafter [36]. The cycle of expression ends with the L proteins, which include the structural proteins needed to create new virions. The dynamics of our model should agree with the timeframe of infection [32,36–38], as well as being computationally feasible. S1 Fig shows the results of a deterministic version of the model using ordinary differential equations (ODEs), which improves upon a previously published model [32]. Here, we use the more standard Hill [39] terms for gene regulation (see Methods and Eq 1), whereas the previous model used only mass action kinetics. Simulation results of the model using the stochastic Gillespie Algorithm but without APOBEC mutations, are shown in S1 Fig, which shows the average of 10 simulations for levels of DNA, mRNA and virion. As expected, these curves are similar to the deterministic simulation results of S1 Fig.

Our model tracks the number of healthy and mutated genomes, gene transcripts, and proteins in a typical cell. It allows for the possibility of *trans-*acting products, i.e., products from one genome that may impact a different genome. HSV-1 can form a number of replication centers (RC) during infection [40,41], so we assume that products from one RC may interact with another during infection. During transcription events we allow A3-related mutations to occur stochastically and separately for the IE, E, and L genes. We implement this by defining a probability of A3 mutation, where a “successful” event causes a non-sense mutation in the target gene (IE, E, or L) with the corresponding viral genome then being tagged as non-functional. An “unsuccessful” event includes the situations wherein: 1. no mutation occurred, or 2. a synonymous mutation occurred. Due to the fact that there are very few overlapping open-reading frames (ORFs) in the HSV-1 genome [42], we do allow for the case that a genome defective in IE genes, for example, may still transcribe other temporal genes (E, L) normally. We define “functional virions” (FVs) as those virions with an unmutated genome in the nucleocapsid capable of creating plaque-forming-units (PFU), as opposed to “non-functional virions” (NFVs) which contain at least one mutated gene. We assume that even though host cells may be susceptible to a NFV, the infection will not progress.

### Mutations early in infection affect the virus more than in later stages

**Fig 2:**
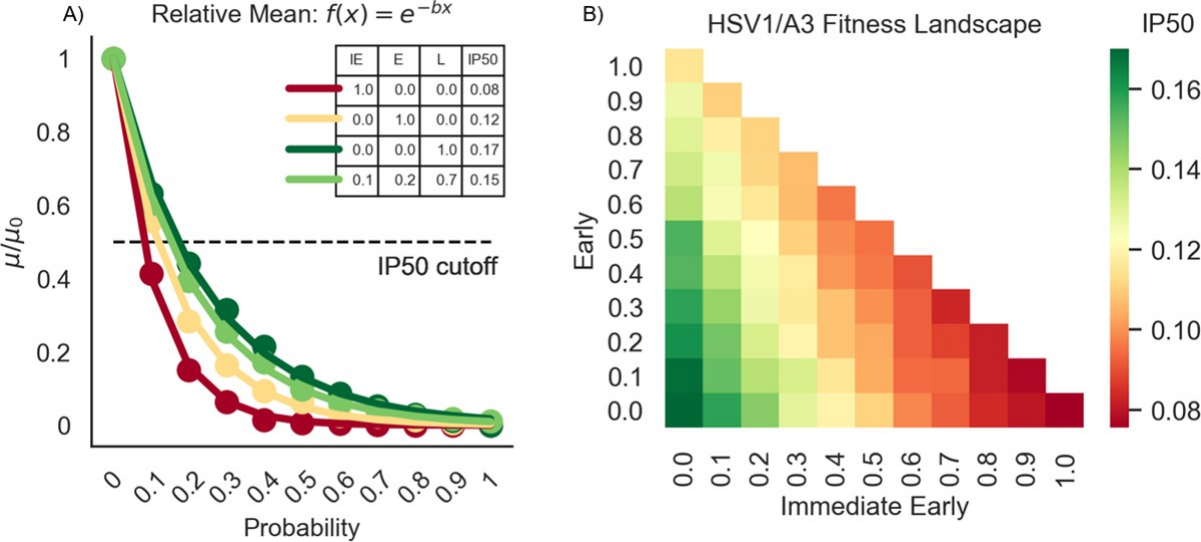
Non-linear least squares analysis and subsequent fitness landscape of HSV-1 IE, E and L genes show that IE and E genes are more vulnerable to A3 mutation than L genes. A) Relative mean, defined as the ratio of means (μ) from each increasing mutation probability versus the mean when the mutation probability is zero (μ_0_). For each baseline probability, these values are fit to an exponential curve f(x) = e^-bx^ using non-linear least squares. The numbers for each stratification in the legend denotes the half-maximal inhibitory probability (IP50) for that stratum. The IP50 is analogous to the half-maximal inhibitory concentration–it measures how high of a mutation probability we need to reduce μ/μ_0_ by 50%, calculated by $\frac{\ln\left(2\right)}{b}$. Shown here are the strata: IE/E/L = .1/.2/.7 (light green), IE/E/L = 1/0/0 (dark red), IE/E/L = 0/1/0 (yellow), and IE/E/L = 0/0/1 (dark green). B) A heatmap of all strata. The gradient up the x-axis denotes a higher proportion of pressure on IE genes. The gradient up the y-axis denotes a higher proportion of pressure on E genes. Finally, the gradient down both the x-axis and y-axis denotes a higher proportion of pressure on L genes. The intensity of each color reflects the IP50 for that stratification–redder indicates a higher A3 pressure (low IP50), and greener indicates a lesser A3 pressure (high IP50). Note that the line colors in A) correspond to the heatmap intensities of those strata in B).

We performed simulations at different multiplicities of infection (MOI) typically used in cell culture infection studies. In our model, the probability that A3A/B mutates during a transcriptional event (Fig 1A and 1B) is defined in two steps. We first define an overall baseline mutation probability that determines whether or not a mutation from A3A/B occurs (or is synonymous) or if it is nonsynonymous, and then further define three individual weights that separate this baseline probability into individual probabilities for each temporal category (IE, E or L) to account for the relative accessibility of ssDNA in each category, which will depend on the number of genes and their lengths, among other factors. In the first instance the relative weights were assigned approximately proportional to the number of genes in each temporal category (IE = 0.1, E = 0.2 and L = 0.7). Note that these three weights lead to only two free parameters—the third weight is defined implicitly given that the sum of the three must equal 1 (here, 0.1 + 0.2 + 0.7 = 1). Below, we refer to any particular triplet set of weights as a “stratification”. From here, we investigated the consequences of varying levels of A3 mutations by evaluating baseline mutation probabilities over a range between 0.1 and 1.0. Thus, for example, when the baseline probability = 0.1, the IE, E, and L genes have mutation probabilities of .01, .02, and .07, respectively. Similarly, when baseline = 0.2, IE, E, and L genes would have mutation probabilities of .02, .04, and .14, and so on. We ran 1000 independent simulations for each baseline probability. S2 Fig shows histograms of the functional virion (FV) count for baseline probability values between 0 and 1 at intervals of 0.1, and Fig 2 shows the relative mean of the number of FVs in the simulations, where relative mean refers to the mean of each simulation divided by the mean of the “wild-type” (WT), i.e., the simulation where no A3-related mutations occur. We also measured the FV percentage (FVP), defined as the fraction of simulations wherein at least one FV was created during infection, since, in the cases of low MOI, one FV unit may be enough to initiate infection in other host cells (S3 Fig) [43], though substantially more than one FV unit may be required for ensuring propagation in the face of stochastic effects.

We modeled the decrease in FVs using exponential decay (Figs 2A and 2B and S3 Fig), and we define the half-maximal inhibitory probability (IP50), an analog of the half-maximal inhibitory concentration (IC50), of each curve as the point on the x-axis where the curve has reached half the maximum (dotted line in Fig 2A). A low IP50 indicates a steeper curve and therefore greater APOBEC impact; in other words, a lower A3 mutation probability is enough to decrease the production (of functional virions) at a much faster rate. The IP50 for the relative mean shown by the light green curve of Fig 2A corresponds to one specific stratification of the baseline probabilities (approximately proportional to the number of genes in each temporal category: IE = 0.1, E = 0.2 and L = 0.7). We next sought to evaluate different possible different stratifications (between IE, E and L). Fig 2A also shows the curves for the three extreme parameter combinations in which the mutational pressure is entirely on the IE, (IE = 1, E = 0 and L = 0), E (IE = 0, E = 1 and L = 0), or L (IE = 0, E = 0 and L = 1) genes. We proceeded by systematically testing all combinations of stratifications of IE, E, and L and performing simulations for each, as we did in Fig 2A, to obtain an estimate for IP50 (A3A/B impact) for each of those stratifications. The results are shown in the heatmap of Fig 2B where the red-filled squares indicate the highest A3A/B impact (small IP50) and the green entries indicate the lowest impact (high IP50). The resulting IP50 values for the three extreme parameter combinations of Fig 2A correspond to each of the three corners in Fig 2B. From Fig 2B we observe that A3 has the biggest impact on the virus at the earliest stages of infection, particularly IE but also on E, and has the least effect on late genes. Because we are surveying all possible combinations, this heatmap can also be viewed as a fitness landscape. In particular, any ancestral viruses would have been situated somewhere within this fitness landscape and also would have been subjected to evolutionary pressures accordingly. Because A3 has the highest impact on IE and E gene expression, this suggests that there will be stronger evolutionary pressure to reduce the impact of APOBEC on the IE and E genes.

Since DNA can become single-stranded during replication, we included an additional mutation probability to represent replication-associated mutations that is independent of transcriptional events. The commonly accepted model of HSV-1 replication involves an initial round of bidirectional theta replication which serves as a precursor to rolling circle replication. HSV-1 DNA is also found as branched non-linear DNA replication intermediates that may include some classical replication forks [44]. S4 Fig shows the effect of different mutational replication probabilities whilst keeping all transcription mutation probabilities equal to zero (see Fig 1A and 1B). Since it has been previously shown that APOBEC primarily deaminates the lagging strand during replication [45], we also assume that mutations that occur during replication only affect the template strand and not the nascent strand. Consequently, mutations during replication will mutate one genome copy, while leaving the other unmutated. Theta replication features two replication forks. Since it is not known whether A3A/B targets both strands, we next considered the case where mutations occurring during replication targets both strands (S4 Fig). In this situation, we get the same IP50 values as in the case where we stratify the probability as IE = .1, E = .2, and L = .7 (Fig 2). However, this latter analysis presupposes only theta replication, whereas the evidence suggests there is only limited theta replication as a precursor to rolling circle replication [44]. We fit the resulting curves, corresponding to the decrease in FVs as a function of mutation probability, into two separate functions–an exponential decay function in the case of the relative mean (IP50 = 0.28), and a linear decay function for FVP (slope = -0.51, S4 Fig). Although we see a decrease in the number of FVs, the rate of decrease is far less than that of transcription-related mutations. This is to be expected since the number of genomes APOBEC mutates would presumably be very small compared to the amount of DNA replication that occurs, given that DNA replication occurs exponentially [46].

### High MOI may compensate for mutations early in HSV-1 infection

**Fig 3:**
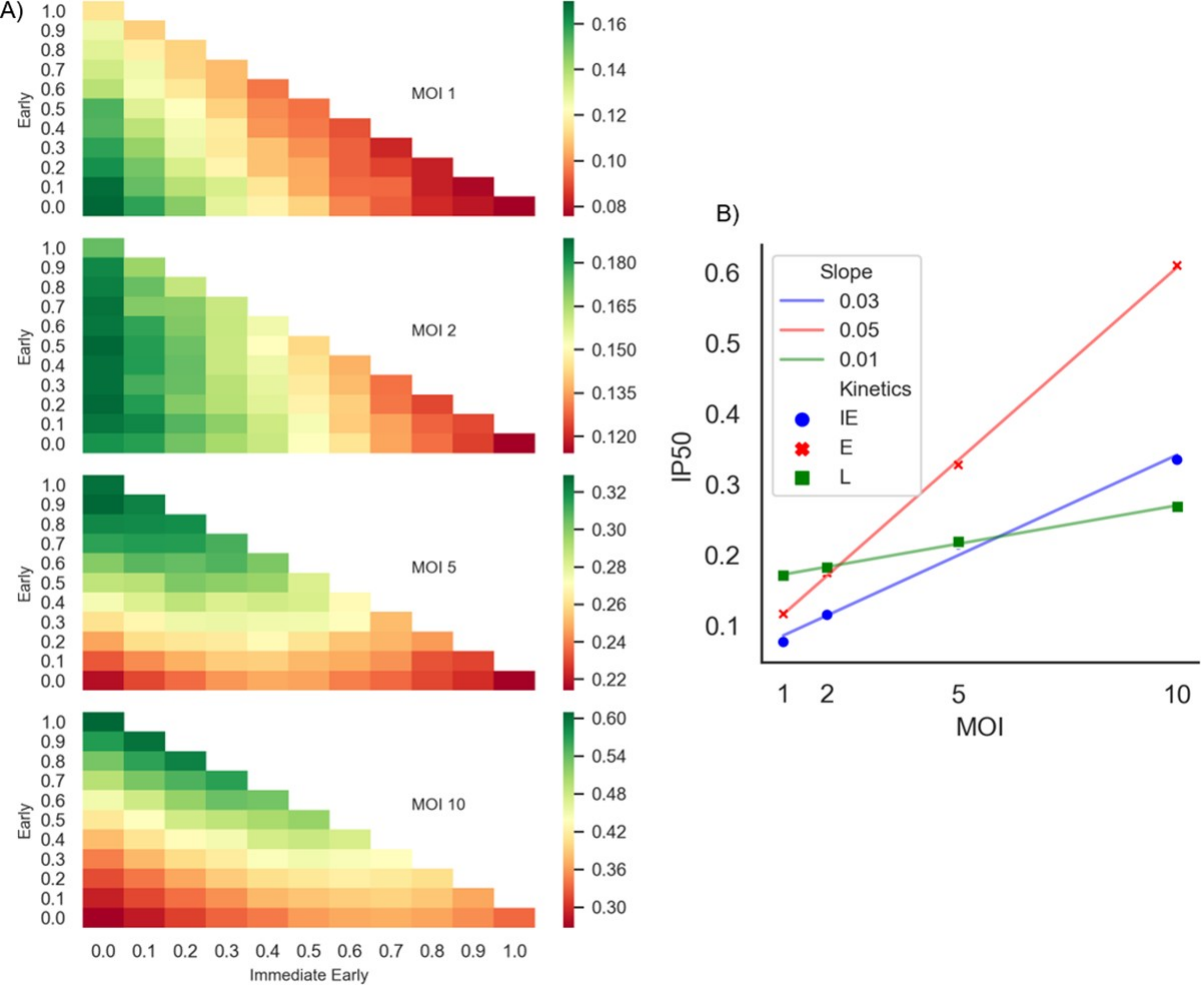
Increasing MOI alleviates pressure on IE and E genes, while A3 pressure on L genes remains more constant. A) Heatmap for MOI = 1 (top) as described in Fig 2B is compared to heatmaps for MOIs of 2,5 and 10. The intensity of colors represents the IP50 values calculated for each of the MOI categories. The relative A3 related pressures on E genes are alleviated as we increase the MOI, while there is nominal increase in the L genes. For each MOI, a different scale is used to highlight the relative IP50 changes. B) A regression of IE (blue), E (red), and L (green) describing how much the IP50 changes as we increase MOI on the three extreme stratifications. L gene IP50 values has a negligible change, as shown its slope (0.01).

In vitro experiments have previously shown that HSV-1 infection at differing MOI yields differential viral gene transcription rates [26,47]. Using our model, we sought to evaluate whether the virus can mitigate the effects of genome mutations by infecting at a higher MOI. Similar to some other DNA viruses, HSV-1 forms replication compartments in the nucleus, wherein parental genomes become templates for HSV-1 replication and such that only one parental genome is found in each compartment [48,49]. Furthermore, there is a maximal number of ~10 replication compartments that can be formed, independent of the MOI, representing a bottleneck for parallel viral replication [43,49,50]. Considering these studies, we implemented our model with MOIs of 1, 2, 5, 10. As we increase MOI, we see that pressure on E genes is eased, followed by IE genes then L genes (Fig 3A). In other words, the difference in mutational pressure changes most significantly in E genes as we increase MOI, as the IP50 difference is 0.494 between MOI 1 and MOI 10, as compared to 0.258 and 0.098 for IE and L genes, respectively (Fig 3B). These impacts with varied MOI suggest that the increase in parental genomes in the replication compartments compensates for A3 mutations. Regardless of the MOI value, A3A/B mutations during L gene transcription have a smaller detrimental effect. There is a much lower difference in weights when we increase the stratum towards L genes (Fig 3), whether we have a MOI of 1 or 10. This supports a bottleneck effect due to the limited number of replication compartments. However, the impact of higher MOI is more pronounced on IE/E genes than for L genes, as evidenced by steeper regression lines for IE and E genes than for L (Fig 3B). For L genes, the impact of A3 changes much less if we increase MOI, whereas the A3 impact changes more for IE and E genes as we increase MOI.

The higher MOI may be allowing functional genomes to compensate for deficient genes of non-functional genomes, which may not be possible for MOI = 1. To test this hypothesis, we ran simulations with MOI = 2, wherein the infection was initiated with one functional and one non-functional genome, separately evaluating cases where non-functional genomes have mutations in one of the IE, E or L gene categories. These simulations were compared to typical MOI = 2 simulations having two functional genomes. We compared the distribution of FV numbers for probabilities of mutation in the range 0.1–1 (as in Fig 2A). For each probability of mutation, we performed an independent t-test comparing the two distributions of the number of FVs and combined the t-test P values using the Fisher method (S1 Table). We then repeated the experiments with MOI = 10 using 5 non-functional and 5 functional genomes, compared to typical MOI = 10 with 10 functional genomes. In all cases, the results showed that there is no difference between wild-type infection or infection where half the genomes are non-functional. Thus, functional genomes compensate for non-functional genomes, regardless of whether the deficiency lies in the IE, E or L genes. Furthermore, these results suggest that selective pressures on the IE and E genes occur at MOI ~1, as in the case of the IE protein ICP0 [51,52].

### Sequence analysis of A3 hotspots indicate that earlier temporal genes are under greater evolutionary pressure from A3 than late genes

A3s evolved to deaminate at specific hotspot DNA motifs, for example TC for A3A/B. As deamination targets, viral sequences may have evolved to reduce the number of viable hotspot targets, or have had these hotspots mutated away, which in turn may limit the negative effects of A3 on the virus. In part due to codon degeneracy, viral sequences may evolve to have fewer A3 hotspots while remaining viable. In order to analyze HSV-1 genes, we used the Cytidine Deaminase Under-representation Reporter (CDUR) program that we previously developed [18]. CDUR compares the number of hotspots in a viral coding sequence with a null distribution of sequences that preserve the amino acid sequence but have shuffled nucleotides at the third codon position (the “n3” module). CDUR not only quantifies hotspot motif under-representation, but also how likely transition mutations at hotspots, such as C>T, are to cause nonsynonymous amino acid changes as opposed to synonymous changes, a measure we termed mutational “susceptibility” [18].

In a previous analysis of the gammaherpesviruses EBV and KSHV, we reported that genes under evolutionary pressure from A3s have hotspot under-representation concurrent with high susceptibility of retained hotspots to amino acid changes. High susceptibility is defined by hotspots that have a higher than expected probability of causing an amino acid change. We hypothesized that high susceptibility would be a consequence of depleting hotspot motifs at synonymous positions, mostly leaving hotspots at non-synonymous positions that cannot be removed without causing amino-acid changes that compromise protein function [19].

**Fig 4:**
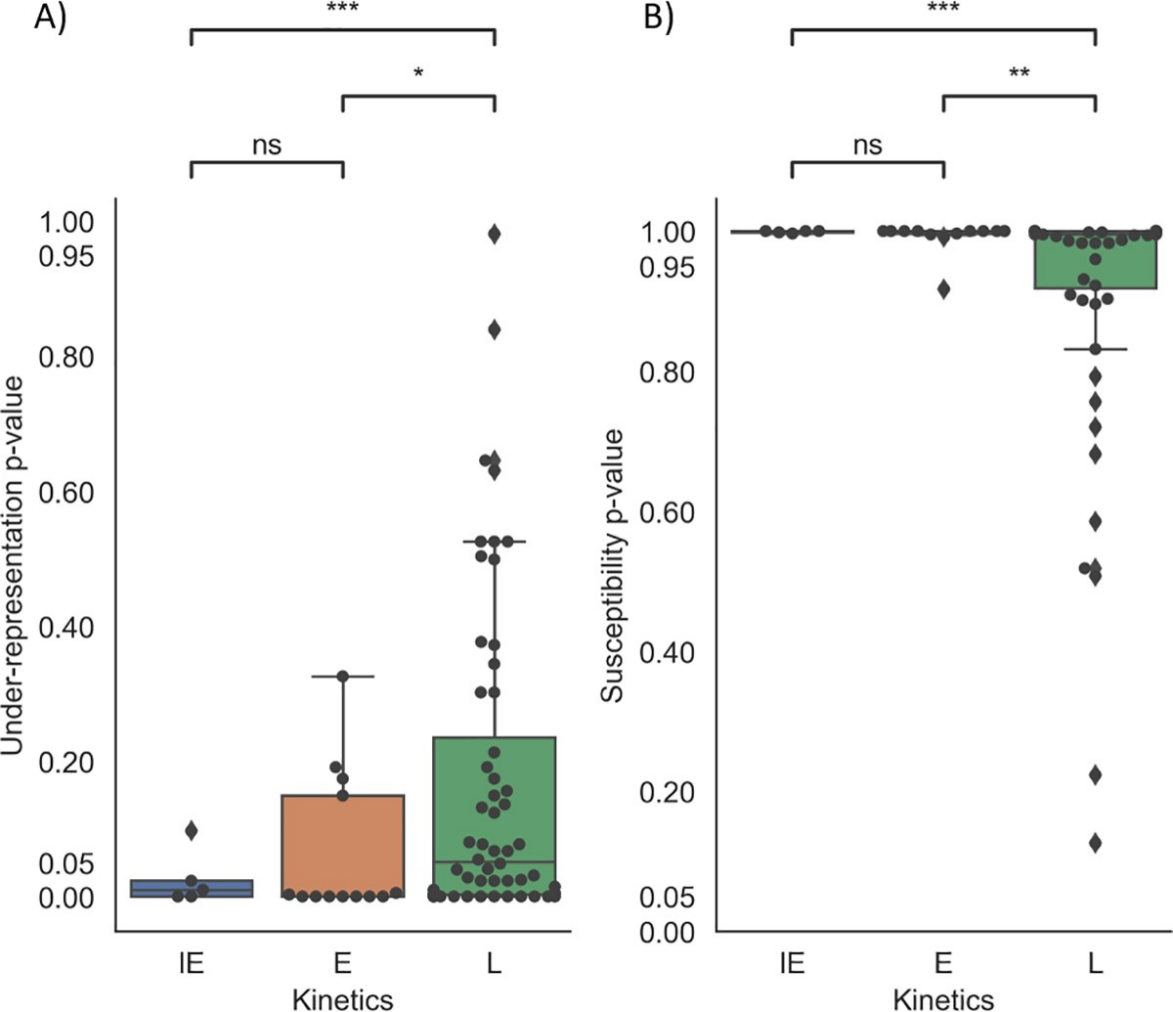
CDUR analysis on HSV-1 strain 17 shows that IE and E genes are significantly under more A3-related evolutionary pressure relative to L genes. A) Under-Representation and B) Susceptibility results for HSV-1 reference strain 17. We performed a pairwise comparison the IE, E and L genes using Welch’s t-test. There was no significance between IE and E under-representation, but there were significant differences between IE/L and E/L genes. *: P< 0.05, **: P<0.01, ***: P<0.001, ns:not significant. CDUR results for all genes were subjected to FDR correction using the Benjamini-Hochberg method.

Here we evaluated TC motifs in HSV-1 using CDUR, since A3A and A3B deaminate at these motifs and localize to the nucleus, where HSV-1 transcription and viral DNA replication occur. Furthermore, previous studies have implicated A3B in restriction of various herpesviruses [19–22]. We considered a gene under-represented if, after a false discovery rate (FDR) correction, it had a *p*-value of < 0.05, and susceptible if it had a post FDR correction *p*-value of >0.95, in other words, if the *p*-value is on the rightmost tail of the null distribution for susceptibility, as one would expect if it is under selection (see S2 Table and Methods). In Fig 4, we analyzed the genes of HSV-1 reference strain 17 (NC_001806), comparing IE, E, and L genes (blue, orange, and green boxes, respectively). IE and E genes have lower under-representation of TC hotspots than the L genes (Fig 4A, Welch’s t-test, P < 0.0004 for IE and L genes, P < 0.0233 for E and L genes), and they both have a higher susceptibility than L genes (Fig 4B, P< 0.0001 for IE and L, P < 0.0028 for E and L). Together, these two results are consistent with stronger evolutionary pressure from A3A/B on the IE and E genes, as predicted by our model (See S3 Table for a list of genes and their kinetic class).

In addition, we wanted to analyze a herpesvirus whose host lacked any A3 restriction, as a negative control. We chose Ostreid herpesvirus 1 (OSHV-1, accession NC_005881), which has an invertebrate host (oyster) that lacks proper APOBEC genes, as well as Marek’s Disease Virus, also known as Gallid Herpesvirus 2 (GaHV-2) and similarly analyzed their genomes using CDUR [53]. In OSHV-1, few of the genes (<12 of 124 genes analyzed) of the genes analyzed show any under-representation or susceptibility to A3 evolutionary pressure. For GaHV-2, 4/85 (~5%) and 8/85 (~9%) of genes analyzed showed significant under-representation and susceptibility, respectively. We also wanted to assess if there is a difference between IE, E and L genes as above. To do this, we classified GaHV-2 homologs based on gene descriptions or a BLASTp search in NCBI (GaHV-2 NC_002229 and HSV01 NC_001806). Those with no clear homolog were categorized as “unknown.” We determined that there was no significant difference in under-representation or susceptibility between IE, E and L genes (S5 Fig). To determine whether the observations for the reference strain 17 hold for other HSV-1 genomes, we repeated the analysis on a previously published dataset of 25 HSV-1 genomes [54] (S2 Table). Consistent with the results of Fig 4, all genomes showed no significant difference between under-representation or susceptibility between IE and E genes. All genomes show significant differences in susceptibility for both IE and E genes against L genes, with 24/25 genomes showing a significant difference in under-representation between IE and L genes. There were only 4/25 genomes that showed a significant difference in under-representation between E genes and L genes (S6 Fig).

### C-to-T mutation analysis in extant strains and clinical isolates of HSV-1

**Fig 5:**
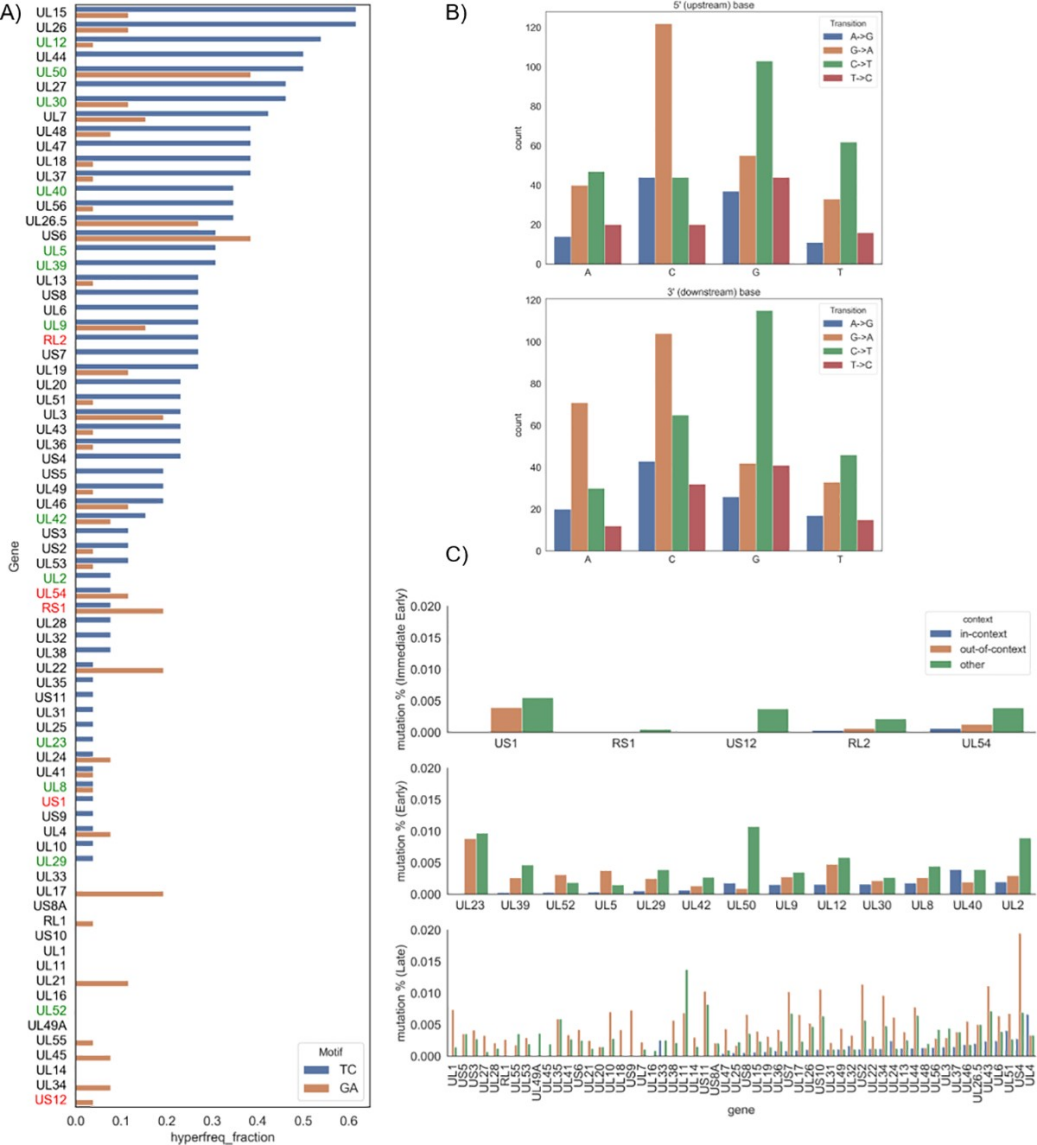
Hypermutation and SNV analysis shows that IE genes yield fewer mutations during HSV-1 infection. A) *hyperfreq*, a Bayesian analysis tool to determine hypermutation, was used on the 26-genome alignment from [54]. The hyperfreq_fraction measure describes the fraction of analyzed genomes that were shown to be hypermutated in TC (blue) or GA (orange) motifs. This was run for each gene in HSV-1. Red is for IE genes, green for E genes, and black for L genes. B) SNVs determined from 10 clinical samples using a threshold for allelic fraction using bcftools (see Methods). Shown are transitions: A>G in blue, G>A in orange, C>T in green, and T>C in red. The x-axis in the top plot shows the 5-prime context for a given SNV, while the bottom shows the 3-prime context. C) The SNV counts were mapped to each gene and normalized by the gene length to determine the mutation % for Immediate Early genes (top), Early genes (middle), and Late genes (bottom).

Similar to our previous work [19], we also analyzed extant strains to determine if there are specific areas in the genomes more susceptible to mutations from APOBEC. We looked at 26 passaged strains of HSV1 sequenced and aligned by [54]. For each site in the alignment, a single nucleotide variant (SNV) was called if at least two sequences have such a variant. S7 Fig shows the allelic fraction of C>T (G>A) transitions in A3A/B contexts (TpC/GpA) and in non-A3A/B contexts. The figure shows that in context transitions are not concentrated in any particular region, and the allelic fraction of in context mutations is significantly less than out of context transitions (P-value < 1.71 e-74). We also utilized the *hyperfreq* program [55] to determine the extent of hypermutation of each gene in either TC or GA motifs, which were examined separately. This program uses a Bayesian approach to determine if the ratio of in context mutations to out of context mutations is statistically significant (see Methods). We performed this analysis by demarcating the whole-genome alignment to be consistent with coding sequences (CDS) described for HSV-1 strain 17 (Genbank: JN555585, obtained from [54]). Only one IE gene showed hypermutation in TC hotspots in more than 10% of the genomes (*RL2*), and only one IE gene show hypermutation in GA hotspots in more than 10% of the genomes (*RS1)*. More than half of the E and L genes show hypermutation in the 26 strains (Fig 5A). Even though few genomes showed hypermutation in nearly all the IE genes, there was no statistical difference in these fractions for IE and E genes, IE and L genes, and E and L genes.

While the previous data describe what occurs in extant strains, we also looked at 10 clinical isolates taken from the NCBI database, sampled via oral lesions [56], and aligned them using HSV1 strain 17 as a reference (NC_001806). We looked at single nucleotide transitions across all 10 isolates and, as expected, we observed that most transitions across the genome occurred in CpG motifs (Fig 5B). We further counted the SNVs found in the CDS of each gene, normalized by the length of that gene (Fig 5C). The IE genes had the lowest in context mutation rate (mean = 0.00019) which was much lower than E (mean = 0.00127) and L (mean = 0.00091) genes (Table 1). This suggests that while A3A/B mutations may be occurring during infection, the IE genes have undergone the most extensive depletion of A3A/B hotspots to avoid mutations during infection.

**Table 1:** Total SNV differences between IE, E, and L genes.

	Mean	Std	Pvalue*
**Immediate Early**	0.00019	0.00029	IEvE = 0.0428
**Early**	0.00127	0.00106	EvL = 0.3326
**Late**	0.00091	0.00122	IEvL = 0.1992

*p values were calculated using a student’s t-test.

### Two stage kinetic model and sequence analysis of human Polyomavirus

We next sought to determine if the modeling and genome analysis results for HSV-1 also applied to a different dsDNA virus with a similar lytic program of sequential temporal expression (S1 Fig). Polyomaviruses (PyV) have much smaller genomes than herpesviruses. For example, the well characterized SV40 virus has a genome slightly larger than 5Kb with 5 genes. The PyV lytic life cycle has two stages: an early stage (E) where both the large-T and small-T antigens are expressed, and a late stage (L) where the structural proteins VP1, VP2, and VP3 are expressed. As in the case of HSV-1, there is evidence for self-repression of E genes, with miRNAs from the late promoter and L gene expression also reducing E gene expression [4]. Immediately after polyomavirus infection, early mRNAs accumulate, while late mRNAs accumulate more slowly such that at approximately 12 hours after infection, the early-late RNA ratio is approximately 4 to 1. At 12–15 hours post-infection, viral DNA replication begins. Here, late mRNAs begin to accumulate rapidly while early mRNAs accumulate at a slower rate, and by 24 hours post-infection, the early to late RNA ratio is as low as 1 to 50. This early-late switch is dependent on viral DNA replication, which in turn changes transcriptional elongation and RNA stability [57]. This timeframe reflects infection kinetics of Mouse Polyomavirus in mouse fibroblast culture. BK polyomavirus has been shown to have a much longer timeframe in renal proximal tubule epithelial cells; E gene expression was detected 12–24 hpi with a continued increase 24–36 hpi. DNA replication and L gene expression appeared to begin 36 hpi [58,59].

**Fig 6:**
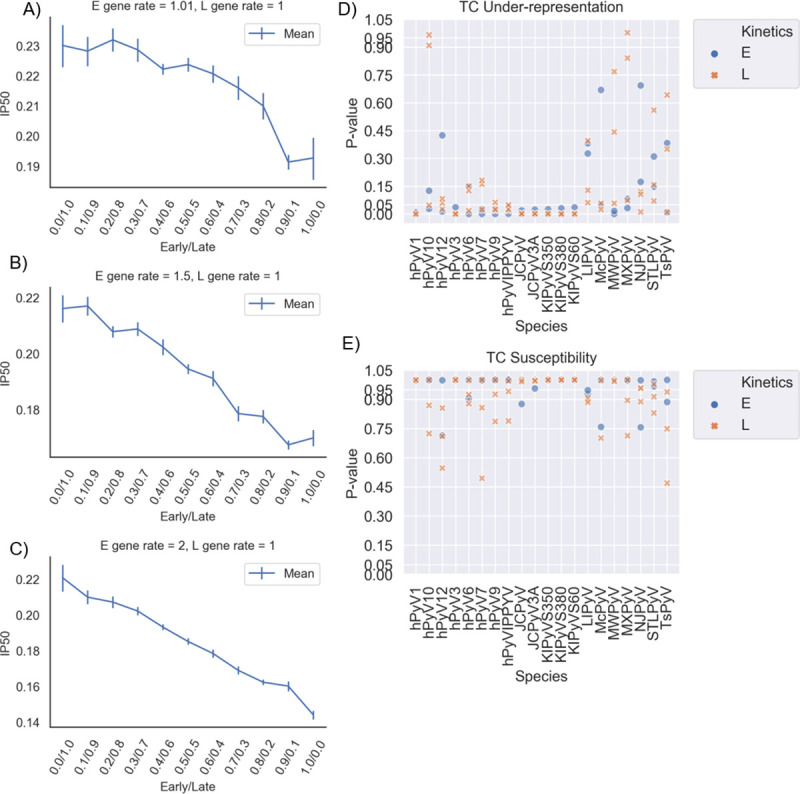
Results from two-step PyV Gillespie model and subsequent CDUR results show a similar pattern to HSV-1 of A3-related evolutionary pressure, under-representation, and susceptibility. A-C) Each plot represents particular gene expression rates for E and L expression. Blue lines represent the IP50 corresponding to the weights *b* from the least squares results when we fit the relative means of the simulations into the exponential decay equation f(x) = *e*^*-bx*^. Note that, as opposed to the HSV-1 case that had three categories of gene kinetics, here a two-dimensional graph for IP50 was sufficient. D-E) CDUR *P*-values for 20 NCBI RefSeq genomes (see Methods) for E and L genes. (D) shows the values for under-representation, while (E) shows susceptibility for TC hotspots. Since there are only 5 genes in each genome, the P values were not FDR corrected.

We adapted our stochastic model to have only two stages (see Methods). Given the discrepancies of timeframes stated above, and given the fact that the stated timeframe was only given for BK polyomavirus, to account for differences in the timeframes, we varied E ($\gamma_{E_{m}}$) and L ($\gamma_{L_{m}}$) gene expression rates across a broad range of plausible values as ${(\gamma }_{E_{m}},\gamma_{L_{m}})=(2,1),(1.5,1),(1.01,1)$. In addition, we systematically varied the Hill and half-saturation coefficients. As for HSV-1, we considered all combinations of mutation probabilities for the E and L genes, which we will call μ_E_ and μ_L_, respectively. Fig 6A, 6B and 6C shows the simulation results for each value of μ_E_ and using the gene expression rates stated above. Since there is only one degree of freedom here (because μ_E_ + μ_L_ = 1), each plot shows the A3 impact (y-axis) for each value of μ_E_, with μ_L_ defined implicitly as 1- μ_E_. In all cases (Fig 6A, 6B and 6C), the impact of A3-related mutation increases as the E mutation rate increases. In addition, simulations that varied the Hill coefficients and half-saturating constants revealed that mutational pressure on E genes is greater than for L genes for a wide range of parameters (S8 Fig). To validate the results of our HPyV model, we applied CDUR to analyze a set of 20 human Polyomavirus (HPyV) genomes obtained from Genbank (Fig 6D and 6E).

To test for a general pattern of a significant difference of under-representation and susceptibility between E and L genes, we considered the vector of the difference of the means of E genes and L genes (**μ**_**L**_**−μ**_**E**_ in the case of under-representation and **μ**_**E**_**−μ**_**L**_ in the case of susceptibility) and then considered a 1-sample t-test against a mean of 0. There was no significant difference when it came to under-representation, but there was a difference in susceptibility (P < 0.0092). The alphapolyomaviruses had the fewest number of under-represented genes, supporting a recent report by [60] (S1 Data). We also used 11 bird polyomaviruses as a negative control. The fraction of under-represented E genes and susceptible E genes is higher in the HPyV dataset than in the bird PyV dataset, as well as L gene susceptibility (S1 Data). In the bird dataset, there was no significant difference between E and L gene under-representation and susceptibility.

## Discussion

In order to study the effect of the A3 cytidine deaminases on a well-characterized dsDNA virus, we developed computational models of the HSV-1 and polyomavirus lytic life cycle. A3 has previously been shown to interact with both herpesviruses [17,19–21] and polyomaviruses [61]. We extended a previous model of the HSV-1 lytic cycle [32] by adding regulatory steps to bring the model dynamics closer to those observed experimentally. In particular, we included regulatory interactions including autoregulation of IE genes such as ICP4 [62–64] and an independent regulation from the onset of L gene expression [4], implemented as two additive Hill functions for IE gene expression; or induction by E genes, such as ICP22, of both E and L genes [65]; as well as induction of L genes as onset of DNA synthesis with additional IE proteins [66,67], implemented as a product of Hill functions for L gene expression. We first defined the system as an ordinary differential equation (ODE) model and fit parameters to the observed timeframe of IE, E, and L gene expression, DNA replication and virion production [32,36–38]. Using the interactions from the ODE model, we then implemented a stochastic version of the model based on the Gillespie Algorithm (Fig 1).

A stochastic model is particularly appropriate for the case of low MOI, as with HSV-1 [43], and where there are low molecule numbers generally [68]. A stochastic model allowed us to associate (probabilistically) an APOBEC mutation with each transcription or replication event where ssDNA is made available. For transcriptional events, different levels of overall mutation probability were defined and further stratified into relative weights for IE, E and L genes respectively. The model showed that mutation events occurring at earlier infection stages, and particularly in IE genes, have a more detrimental impact than in L genes. The systematic evaluation of all mutation weight combinations for IE, E and L genes (Fig 2) can also be viewed as a fitness landscape for the virus in the face of APOBEC mutation, suggesting that there has been, and continues to be, strong evolutionary pressure on the virus to evolve to avoid mutations in E and particular IE genes.

If we increased the MOI, then APOBEC impact was significantly decreased for IE or E genes, but not L genes. This agrees with our current understanding of the high rate of HSV-1 replication, by the time L genes are expressed, genomic mutational defects from A3 will lessen due to the number of genomes present and possible genomic recombination. When the MOI is high, even mutations that occur during IE and E transcription can be compensated for by *trans* effects. We speculate a low MOI infection will allow for rapid genomic diversification. Although DNA viruses generally replicate with greater fidelity than RNA viruses, which show mutation rates of 10^−4^ to 10^−6^ errors per nucleotide per infection cycle, HSV-1 mutates at a nontrivial intermediate rate of 10^−7^ errors per nucleotide per infection cycle [69,70], and perhaps bottleneck effects during infection may act to increase diversification further [70].

We previously used CDUR [18] to assess viral genomes for evidence of APOBEC-mediated evolutionary pressure, in particular by identifying genes having significant hotspot under-representation and depletion of hotspots at codon positions leading to synonymous changes [19]. Recent work examined the footprint of APOBECs on dsDNA viral genomes [60] based on under-representation of viral genes from HSV-1 and HPyV. Under-representation was reported in some genes in HSV-1 and various polyomaviruses. The method used is less conservative than CDUR in that it allows rearrangement of the CDS at the codon level, which may result in biologically unfeasible protein structures. CDUR methods preserve the underlying amino acid sequence with limited effects on codon bias and codon pair bias, which have been shown to affect viral fitness via defective translation rates [30,71].

To validate our model, we analyzed the benchmark strain 17 HSV-1 genome [54] using CDUR. We found, as predicted, that IE and E genes are under greater evolutionary pressure to avoid mutations than L genes. In contrast, EBV, which we analyzed previously [19] and human cytomegalovirus (CMV, S5 Fig) do not have the same pattern of under-representation as HSV-1, which may be explained by the stronger restrictive effects of the EBV BORF2 gene when compared to equivalent HSV-1 gene, ICP6. Since virulence was not affected in ICP6-null infection [22], our results suggest that sequence evolution of IE and E genes may be why the function of ICP6 could have been rendered redundant. There may also be a difference in stem-loop structures, which has been shown to affect APOBEC binding and mutation [72]. When we extended the analysis to 25 HSV-1 strains, the results were similar to what we found for HSV-1 strain-17. Certain key proteins, such as ICP4, were consistently under-represented for TC motifs, suggesting that HSV-1 may have evolved to combat APOBEC3 mutations by reducing the number of binding motifs, without affecting the underlying amino acid sequence. We also compared Essential vs Non-essential genes in HSV1 strain 17 and found statistically significant differences between these (S9 Fig). This result suggests experiments where one might measure the extent of virus attenuation in an engineered HSV-1 mutant, whose essential IE genes are enriched for A3A/B hotspots. We further evaluated whether under-representation and susceptibility correlated with the distance from the nearest origin of replication using HSV-1 strain 17 (NC_001806) as a reference, but we found no such correlation for under-representation (Pearson r = -0.0911, P = 0.4402) or susceptibility (Pearson r = 0.2017, P = 0.0848). Because HSV-1 has a particularly high GC content when compared to other DNA viruses, we chose the “n3” method that corrects for GC content, as not correcting can have a biasing effect [30]. Our results also suggest that nascent DNA may be used as templates for transcription, since APOBEC-mediated evolutionary pressure on L genes was reduced, though experiments will be needed to confirm this.

While the CDUR results describe the evolutionary footprint of A3A/B on HSV-1 genomes, to determine more recent activity on extant strains, we analyzed a well-curated set of 26 published genomes, as well as looked at sequencing data from clinical isolates. In both cases, there is a clear indication that fewer mutations are occurring on IE genes, which is consistent with our HSV-1 fitness map (Fig 2)–as one moves towards an extreme IE stratum, the decrease in viral fitness reaches its maximum. One potential way for the virus to evolve a strategy against this is to limit the number of TC motifs in those genes. Interestingly, E genes seemed to have more observed variants than expected; more than half of the E genes showed hypermutation in >10% of the 26 genomes analyzed. The L genes showed a large variance in hypermutation fractions (Fig 5A) and number of SNVs observed (Fig 5C). We found that the largest mutation frequency in the 10 clinical isolates were seen in the E genes (Fig 5C).

We extended our modeling and analysis to Polyomaviruses, a dsDNA virus family with a comparable, albeit much shorter, lytic life cycle. Because the temporal dynamics of the polyomavirus life cycle are less well characterized than for HSV-1, we began by choosing parameters that fit the approximate timeframe as follows: 1. E genes are transcribed 6 hpi, 2. L gene transcription and DNA replication begin 12 hpi [57]. We tested a wide range of model parameters to evaluate whether E genes might be under greater APOBEC-mediated pressure than L genes. Similar to our findings for HSV-1, we found that the mean number of virions decreases as we increase mutational pressure in the E genes. Our analysis of 20 distinct HPyV genomes indicated that E genes have significant A3A/B TC hotspot under-representation and high amino acid susceptibility. Taken together, the viral genome has evolved to deplete APOBEC hotspots, up to the point where any further reduction (in non-synonymous codon positions) might lead to non-functionality [19]. Most HPyV genomes were significant for both features, suggesting that the genome has been under evolutionary pressure to remove TC hotspots from E genes more so than L genes. The higher percentage of under-representation and susceptibility in large T antigens (75%) vs small T antigens (60%) is consistent with the greater importance of large T during early infection and its role in many different aspects of the viral life cycle, whereas the small T antigen has been shown to be non-essential for viral replication [73], although it has been implicated in tumorigenesis [4,74]. The large T antigen has been reported to upregulate nuclear A3B [75], another source of evolutionary pressure on the large T antigen. Interestingly, while there was a significant difference in the CDUR analysis in HSV-1 temporal categories for TA and TG motifs (S10 Fig), there was no significant difference between IE, E and L genes in TT over-representation, even though a majority of genes showed strong over-representation in TT hotspots (S10 Fig and S1 Data). TC under-representation (Fig 4A) coupled with TT over-representation suggests a more defined footprint of A3A/B in HSV-1 genes. For HPyV, we found little TT over-representation (S10 Fig) but a large number of genomes had TG over-representation, which implies downstream recruitment of base excision repair, a common feature of many tumor genomes where A3s have been implicated [76–81]. This is interestingly consistent with the findings of [61] which frequently observed TCA:TGA bas changes in VP1, but this also stands in contrast with [82] which suggested that there existed TT over-representation. However, that study only looked at BK Polyomavirus (HPyV1), and they looked at the genome-wide effects using trinucleotide densities, whereas CDUR calculates under-representation gene by gene, and uses a more conservative shuffling approach.

In summary, our modeling of the lytic cycle HSV-1 and HPyV, together with genome analysis, has highlighted an important aspect of virus-host co-evolution between humans and these viruses. The co-evolution has resulted in a unique fitness landscape such that genes expressed early in the lytic cycle appear to be under the strongest A3 pressure. The results of the genomic analyses stand in contrast to other well-characterized herpesviruses such as EBV and KSHV, despite these viruses having a similar pattern of lytic infection. APOBEC pressure on genes expressed during the early part of infection may have constrained these genes, thus reducing their capacity for further evolution and potentially making them good candidates for future vaccines or as therapeutic drug targets.

## Methods

### ODE model of HSV-1 infection

We first developed a deterministic model of the HSV-1 lytic cycle using a system of ordinary differential equations (ODEs), extending a previous model by Nakabyashi et al. [32] by adding the regulatory interactions, taken from [4] as Hill function terms (S4 Table) where the previous model was based purely on mass action kinetics. The ODE system is defined as follows (Eqs 1–8):

$$\frac{dD}{dt}=\alpha_{1}\left(\frac{E_{p}^{n_{E_{p}}}}{k_{E_{p}}^{n_{E_{p}}}+E_{p}^{n_{E_{p}}}}\right)-\alpha_{2}DL_{p}-\delta_{D}D$$ (1)

$$\frac{dI_{m}}{dt}=\gamma_{I_{m}}\left(\frac{k_{I_{p}}^{n_{I_{p_{I}}}}}{k_{I_{p}}^{n_{I_{p_{I}}}}+I_{p}^{n_{I_{p_{I}}}}}-\frac{L_{p}^{n_{L_{p_{I}}}}}{k_{L_{p}}^{n_{L_{p_{I}}}}+L_{p}^{n_{L_{p_{I}}}}}\right)-\beta_{I_{p}}I_{m}-\delta_{I_{m}}I_{m}$$ (2)

$$\frac{dI_{p}}{dt}=\beta_{I_{p}}I_{m}-\delta_{I_{p}}I_{p}$$ (3)

$$\frac{dE_{m}}{dt}=\gamma_{E_{m}}\left(\frac{I_{p}^{n_{I_{p_{E}}}}}{I_{p}^{n_{I_{p_{E}}}}+k_{E_{p}}^{n_{I_{p_{E}}}}}\right)-\beta_{E_{p}}E_{m}-\delta_{E_{m}}E_{m}$$ (4)

$$\frac{dE_{p}}{dt}=\beta_{E_{p}}E_{m}-\delta_{E_{p}}E_{p}$$ (5)

$$\frac{dL_{m}}{dt}=\gamma_{L_{m}}\left(\frac{D^{n_{D}}}{k_{D}^{n_{D}}+D^{n_{D}}}\right)\left(\frac{I_{p}^{n_{I_{p_{L}}}}}{I_{p}^{n_{I_{p_{L}}}}+k_{L_{p}}^{n_{I_{p_{L}}}}}\right)-\beta_{L_{p}}L_{m}-\delta_{L_{m}}L_{m}$$ (6)

$$\frac{dL_{p}}{dt}=\beta_{L_{p}}L_{m}-\alpha_{2}DL_{p}-\delta_{L_{p}}L_{p}$$ (7)

$$\frac{dV}{dt}=\alpha_{2}DL_{p}-\delta_{V}V$$ (8)

All molecular species *i* in our ODE decay linearly at rates δ_*i*_. In the first equation, the amount of DNA that is replicated is dependent on the appearance of E genes. Once the structural proteins are formed, the concatemers are cleaved into genome-length units and packaged into the nucleocapsid at a rate α_2_. IE gene transcripts, denoted *I*_*m*_ (Eq 2), are both negatively regulated by both IE proteins [4,63,64] and the onset of L proteins [4]. These are additive effects since they independently repress IE gene transcription. In addition, we assume a translation rate *β*_*j*_, for each mRNA transcript *j*. All proteins (Eqs 3, 5 and 7) are translated at a rate linear in the mRNA, and, in the case of *L*_*p*_, we include an additional term to reflect the removal of late structural proteins to form virus nucleocapsids. *E*_*m*_ (Eq 4) is regulated by IE proteins, and the corresponding early proteins *E*_*p*_ are responsible for starting replication of the HSV-1 genome. *L*_*m*_ (Eq 6), which are the L gene transcripts, are activated both by IE proteins, and by the accumulation of DNA during replication, together. We reflect this dependence by utilizing a product of Hill terms within Eq 6 [4,66]. Finally, virions *V* (Eq 8) are created when the structural proteins appear, and as the genome is inserted into the capsid to form the nucleocapsid, which in turn will incorporate the envelope and tegument proteins leading to a completed virion.

### Gillespie algorithm and chosen parameters

The Gillespie Algorithm [35] is a widely-used stochastic simulation algorithm that updates the state of the system (counts of each molecule type) based on the reaction rules and their respective rates. The algorithm proceeds in discrete time steps, at each step calculating (a) what is the next reaction to occur, and (b) when it will occur. These are determined as follows: given a state of molecule counts [*a*_1_,…,*a*_*n*_] for reactions [*r*_1_,…,*r*_*n*_] at time *t*, take the sum reaction rate $R=\sum_{i=1}^{n}a_{i}r_{i}$. With this sum, we first choose an elapsed time Δ*t* from an exponential distribution with mean *R*. With it, we move the simulation forward to time step *t+* Δ*t*. To choose the associated reaction, we select (randomly and uniformly) a reaction *i* from the set $\{R_{i}=\frac{a_{i}r_{i}}{R}|i=1,\ldots ,n\}.$ The molecule counts (proportional to concentrations) are then updated to reflect this reaction. We repeat this process until we reach some time threshold *T*. To summarize, the Gillespie algorithm for a set of *n* reactions can proceed as follows (see Fig 1A):

1. Determine the state vector of each species, and determine the reaction rates defined by a vector product of molecule counts [*a*_1_,…,*a*_*n*_] with the corresponding reactions [*r*_1_,…,*r*_*n*_]^T^ to obtain *R* as defined above.
2. Draw two independent samples *R*_*i*_, Δ*t*, one from a uniform distribution and one from an exponential distribution, both as defined above.
3. Adjust the vector of molecule counts according to the chosen reaction and increase time by Δ*t*.
4. Go back to step 1. Repeat until a specified time *T*, or, in the case where the series of partial sums ∑_*i*_ Δ*t*_*i*_ is convergent, a maximal number of steps is reached (in our case, we chose 5000 steps).

There are 16 reactions that can occur in the HSV-1 model (S4 Table), and they correspond to terms in the ODE model of the previous section. To represent A3 mutations, if a transcription event occurs, we generate a mutation according to the given probability for the transcribed gene (IE, E, or L). Once a mutation event occurs, that genome is considered damaged and can no longer transcribe genes of that temporal type (IE, E or L); however, further transcription events can continue in the remaining unmutated genes unless it is mutated again. Replication events may also lead to APOBEC mutations, with a given probability. If a mutation is chosen to occur during replication, then the mutation is assigned to IE, E or L genes using the same proportions (stratification) as for transcription. We ran these simulations for the length of time that corresponded to a normal HSV-1 infection timeframe (~20 hours [36]). All simulations and analyses were run using custom python scripts. The units are described in S6 Table, and the parameter values were chosen to fit our ODE model to the HSV-1 timeframe as previously described and computed in [32,36]: $\delta_{I_{m}},\delta_{E_{m}},\delta_{L_{m}},\delta_{I_{p}},\delta_{E_{p}},\delta_{L_{p}},\delta_{D},\delta_{V}=.0001;$ $\beta_{I_{m}},\beta_{E_{m}},\beta_{L_{m}}=.5,.2,.1;\;\alpha_{1},\alpha_{2}=1,2;$ $\gamma_{I_{m}},\gamma_{E_{m}},\gamma_{L_{m}}=3,1,1;$ $k_{I_{p}},k_{E_{p}},k_{L_{p}},k_{D}=1,.1,1,1;n_{I_{p_{I}}},n_{I_{p_{E}}},n_{I_{p_{L}}},n_{E_{p}},n_{L_{p_{I}}},n_{D}=.5,1,1,1,3,1$. We incorporate the MOI, which is defined as the ratio of virus to infection targets, by initializing the infection with the corresponding number of genomes, i.e., a MOI of 1 saw an initial infection with one unit of DNA, MOI of 10 infects with 10 units, and so on.

For polyomavirus, we also ran Gillespie simulations using the propensities given in S5 Table, which are based on the ODE model (Eqs 9–14):

$$\frac{dD}{dt}=\alpha_{1}\left(\frac{E_{p}^{n_{E_{p}}}}{k_{E_{p}}^{n_{E_{p}}}+E_{p}^{n_{E_{p}}}}\right)-\alpha_{2}DL_{p}-\delta_{D}D$$ (9)

$$\frac{dE_{m}}{dt}=\gamma_{E_{m}}\left(\frac{k_{E_{p}}^{n_{E_{p}}}}{k_{E_{p}}^{n_{E_{p}}}+E_{p}^{n_{E_{p}}}}\right)-\beta_{E_{p}}E_{m}-\delta_{E_{m}}E_{m}$$ (10)

$$\frac{dE_{p}}{dt}=\beta_{E_{p}}E_{m}-\delta_{E_{p}}E_{p}$$ (11)

$$\frac{dL_{m}}{dt}=\gamma_{L_{m}}\left(\frac{D^{n_{D}}}{k_{D}^{n_{D}}+D^{n_{D}}}\right)\left(\frac{E_{p}^{n_{E_{p}}}}{E_{p}^{n_{E_{p}}}+k_{E_{p}}^{n_{E_{p}}}}\right)-\beta_{L_{p}}L_{m}-\delta_{L_{m}}L_{m}$$ (12)

$$\frac{dL_{p}}{dt}=\beta_{L_{p}}L_{m}-\alpha_{2}DL_{p}-\delta_{L_{p}}L_{p}$$ (13)

$$\frac{dV}{dt}=\alpha_{2}DL_{p}-\delta_{V}V$$ (14)

The parameters used were: $\delta_{E_{m}},\delta_{L_{m}},\delta_{E_{p}},\delta_{L_{p}},\delta_{D},\delta_{V}=.0001;\;\beta_{E_{m}},\beta_{L_{m}}=.2,.1;\;\alpha_{1},\alpha_{2}=1,2;\;k_{D},n_{D}=1.$ These parameters were chosen to fit a general timeframe as described in [57], though this particular timeframe is not as exact as the HSV-1 case. We therefore varied $\left(\gamma_{E_{m}},\gamma_{L_{m}}\right)$ to be (2,1), (1.5,1), (1.01,1); the first three cases test for the approximate timeframe that fits [57], and then we vary the parameters to cases where transcription rates of late genes are close to that of early genes. We also varied (*k*_*E*_, *k*_*L*_) = {.1,1,5}×{1,5,10}; (*n*_*E*_, *n*_*L*_) = {1,5,10}×{1,5,10}, where × denotes a Cartesian product, since exact parameters and timeframe could not be found; these different parameters were tested while $\left(\gamma_{E_{m}},\gamma_{L_{m}}\right)$ was constant at (2,1).

We define the relative mean to be the ratio of the mean in a simulation to that of the WT simulation. The functional virion percentage (FVP) is defined as the fraction of simulations that produced a functional virion (equivalent to one plaque forming unit, or PFU). Using non-linear least squares, both the relative mean and FVP results can be fit well to an exponential curve, exp(-b·x) where the single fitted parameter b summarizes the APOBEC impact across the entire range of baseline probabilities. The corresponding IP50 is defined using the half-life of the exponential decay: log(2)/b. Because the sum of the strata (IE+E+L) is 1 and there are only two degrees of freedom, if we define IE and E, then L can be calculated implicitly as 1 –(IE+E), so long as (IE+E) < = 1. Similarly, in the case of HPyV, there is only one degree of freedom. We apply our least-squares method to all combinations of IE and E genes in the case of HSV1 (and E genes in HPyV) for IE = 0,.1, …, 1 and E = 0,0.1, …, 1, in one-tenth increments.

Non-linear and linear least squares were performed in Python using the curve_fit function in the Scipy library and using the LinearRegression class in the sckit-learn library, respectively, and were calculated using custom scripts. Plots were made using Python’s Seaborn library with custom scripts.

### Analyzing APOBEC hotspot under-representation and susceptibility

We utilized the Cytidine Deaminase Under-representation Reporter (CDUR) [18] to determine the sequence evolution measures for the coding sequences (CDS) of the analyzed genomes. CDUR compares the original sequence to a null distribution of 1000 shuffled sequences (the default number). We used the “n3” shuffling method that switches nucleotides in the third position of codons while preserving the underlying amino acid sequence. Importantly, the n3 module also preserves GC content which can be a strong source of bias if not corrected for [30]. To measure hotspot under-representation, CDUR compares the number of motifs in the original sequence to the null distribution, yielding a *p*-value for under-representation. For amino acid changes, CDUR performs a similar test based on the fraction of hotspot transition mutations that are nonsynonymous, yielding a *p*-value for the “replacement-transition-fraction”. We refer to this metric in the main text as the *susceptibility* to nonsynonymous mutations. On each HSV-1 genome, we ran CDUR and then performed a false discovery rate (FDR) correction using the Benjamini-Hochberg method (unless otherwise specified). We also ran CDUR on the coding sequences for HPyV from NCBI accessions NC_001538, JX262162, NC_020890, NC_009238, NC_014406, NC_014407, NC_015150, FR823284, NC_001699, HG764413, EF127907, EF127908, EF127906, NC_034253, NC_010277, NC_018102, JX259273, NC_024118, NC_020106, NC_014361 for hPolyomaviruses, and accessions NC_026141, AF118150, NC_023008, NC_017085, DQ192570, NC_007922, NC_039052, NC_007923, NC_004800, NC_039053, DQ192571 for bird Polyomaviruses. All CDUR results are available in S1 Data.

### Hyperfreq analysis

*Hyperfreq* [55] uses a Bayesian two-context mutation probability ratio to determine if the number of in context mutations are significantly more than out of context mutations. We adjusted the program so that gaps in alignments would be considered as out of context mutations. In addition, we used the built-in consensus builder in *hyperfreq* which makes a consensus using a threshold of 70%. This program was run on TC and GA motifs to determine A3A/B activity on the forward and reverse strands, respectively. Data for the alignment was taken from [54] from the URL szparalab.psu.edu/hsv-diversity/data, as well as the GFF file used to parse the alignment into the proper CDS. Figures relating to these data were created using custom scripts in python.

### Analysis of clinical isolates and resulting single nucleotide variants

Sequencing data was retrieved from the NCBI BioProject database ID PRJNA338014, run IDs SRR8114523, SRR8114524, SRR8114528, SRR8114522, SRR8114526, SRR8114519, SRR8114527, SRR8114520, SRR8114525, SRR8114521. Original sequences came from the Illumina MiSeq platform using paired whole-genome sequencing. Sequences were downloaded from NCBI using the command:

fastq-dump–split-files {}

where {} denotes the SRA ID number. Trimmomatic [83] was then used for quality control using the command:

trimmomatic PE {}_1.fastq {}_2.fastq {}_trimmed_1.fastq {}_unpaired_1.fastq {}_trimmed_2.fastq {}_unpaired_2.fastq SLIDINGWINDOW: 4:30

Bowtie [84] and samtools [85] was used to align the resulting reads to reference genome NC_001806. The commands used were:

bowtie2 -x <reference> -1 {}_trimmed_1.fastq -2 {}_trimmed_2.fastq | samtools sort > {}.bowtie.bam

Finally, bcftools [85] was used to analyze and merge variants using the commands:

bcftools mpileup -Ou -f $REF *bowtie.bam > genotypes.vcf

bcftools call—ploidy 1 -vm -Ou genotypes.vcf | bcftools norm -Ov -f <ref> -d all–

CDS locations were analyzed using only those variants with a depth > 100 and a quality score >50, as well as limited to SNVs that have an allelic fraction of 20–50%. Figures were made using custom scripts in python.

## Supporting information

S1 Fig A) Results of differential equation model which incorporates Hill-type regulatory effects from each kinetic category. B) 10 simulations of the Gillespie algorithm implementation. The thick blue lines indicate the mean. As expected, the timeframe and levels coincide approximately with the ODE model. C) Schematic interactions of Polyomavirus life cycle. The yellow triangles indicate when APOBEC may cause mutations in our model. V = virion, Em = Early mRNA, Ep = Early protein, Lm = Late mRNA, Lp = Late protein.
(TIF)

S2 Fig Frequency plots showing the distribution of the number of virion units across 1000 simulations. Here, we progress through the .1:.2:.7 stratum (denoted 1/2/7). We start by multiplying 0x(.01:.02:.07), then 1x(.01:.02:.07), etc. to simulate increasing APOBEC mutation probability. As we increase the baseline probability, the total number of virion progeny from the simulations gets closer to 0.
(PNG)

S3 Fig Left: Functional Virion Percentage (FVP), defined as the number of simulations yielding an FV greater than 0. For each baseline probability, these values are fit to an exponential curve f(x) = e-bx using non-linear least squares. The numbers for each stratification in the legend denotes the half-life for that stratum. Shown here are the strata: IE/E/L = .1/.2/.7 (red), IE/E/L = 1/0/0 (blue), IE/E/L = 0/1/0 (orange), and IE/E/L = 0/0/1 (green). b) A heatmap of all strata such that the strata sum to 1, i.e., IE+E+L = 1. The L strata can be inferred from the grid by taking L = 1-IE-E, e.g. if IE = .1 and E = .2, then L = 1-.1-.2 = .7. The intensity of each color reflects the half-maximal inhibitory probability (IP50) for that stratification (combination of parameters). The IP50 is analogous to the half-maximal inhibitory concentration–it measures how high of a mutation probability we need to reduce the y-axis by 50%, calculate by ln(2)/b. squares, using one parameter as with relative mean (Fig 2A). Shown here are the strata: IE/E/L = .1/.2/.7 (light green), IE/E/L = 1/0/0 (dark red), IE/E/L = 0/1/0 (yellow), and IE/E/L = 0/0/1 (dark green). Right: A heatmap of all strata such that the strata sum to 1, i.e., IE+E+L = 1. The L strata can be inferred from the grid by taking L = 1-IE-E, e.g. if IE = .1 and E = .2, then L = 1-.1-.2 = .7. The intensity of each color reflects the IP50 for that stratification. Note that the line colors in the left plot correspond to the heatmap intensities of those strata in the right plot.
(PNG)

S4 Fig Results allowing for mutations during replication. A) and B) shows the relative mean, and the right shows FVP for different probabilities (x-axis) assuming rolling circle replication only. C) and D) are the results for when theta replication is modeled as the main method of replication.
(TIF)

S5 Fig Plots for the herpesvirus Cytomegalovirus (CMV), Gallid Herpesvirus 2, and and Ostreid herpesvirus 1 RefSeqs (NCBI accessions NC_006273, NC_002229, and NC_005881, respectively). A) A comparison of IE, E, and L genes for CMV. Kinetics were determined by assessing homology with HSV-1 genes. B) Percentage of genes in GaHV-2 that show under-representation or not, as well as susceptibility or not. C) We compare the different kinetics of IE, E, and L genes in GaHV-2. We see that there are few genes that are significantly under-represented or susceptible, and there is also no difference in the distributions, as determined by Welch’s t-test. b) Ostreid herpesvirus 1 (OSHV-1) analysis of genes for under-representation and susceptibility. Since Oysters lack APOBEC, almost all of the genes show no under-representation or susceptibility from APOBEC3 TC motifs. Values shown are not FDR corrected since p-values were not significant regardless.
(TIF)

S6 Fig Different under-representation and Susceptibility P-values between IE, E, and L genes in 26 HSV1-genomes. (TIF)

S7 Fig The single nucleotide variant ratio at each site of the 26 genome alignment as determined in (Szpara et al, 2014 Journal of Virology). In-context mutations are those SNVs that occurred in a TC>TT or GA>AA context in at least 2 of the genomes at each site. Out-of-context SNVs are those C>T or G>A SNVs that occurred outside of a TC or GA context, respectively. The distribution of SNV ratios across the genome differed significantly between in-context and out-of-context mutations, as determined by a Student’s t-test (P < 1.71 e-74).
(TIF)

S8 Fig Two-step plots using a variety of half saturating constants and Hill coefficients. In each case the results are qualitatively equivalent to those of Fig 6A, 6B and 6C.
(PNG)

S9 Fig Essential vs NonEssential comparisons for HSV-1 genes. A) Under-representation. B) Susceptibility.
(PNG)

S10 Fig TG and TT hotspot over-representation for HSV1 and HPyV. Following the same format for under-representation as Fig 4A and Fig 5D, 5E, plots showing A) HSV-1 TG over-representation (high, rather than low P values) and B) HSV-1 TT over-representation. Following the same format as Fig 6D, plots for C) HPyV TG over-representation. D) HPyV TT over-representation.
(PNG)

S1 Table FVs vs mixed FV/NFV. (DOCX)

S2 Table CDUR analysis on 25 HSV1 genomes. (DOCX)

S3 Table HSV1 genes and their kinetics. (XLSX)

S4 Table Propensities for HSV-1 Gillespie Algorithm. (DOCX)

S5 Table Propensities for two-step Gillespie Model. (DOCX)

S6 Table Units in equations. (DOCX)

S1 Data Tables for CDUR results. (XLSX)
