# Supplementary figures and images for: Mutational pressure by host APOBEC3s more strongly affects genes expressed early in the lytic phase of herpes simplex virus-1 (HSV-1) and human polyomavirus (HPyV) infection
Source: PLoS Pathog. 2021 Apr 30;17(4):e1009560. doi: 10.1371/journal.ppat.1009560 (PMC8115780; doi:10.1371/journal.ppat.1009560)

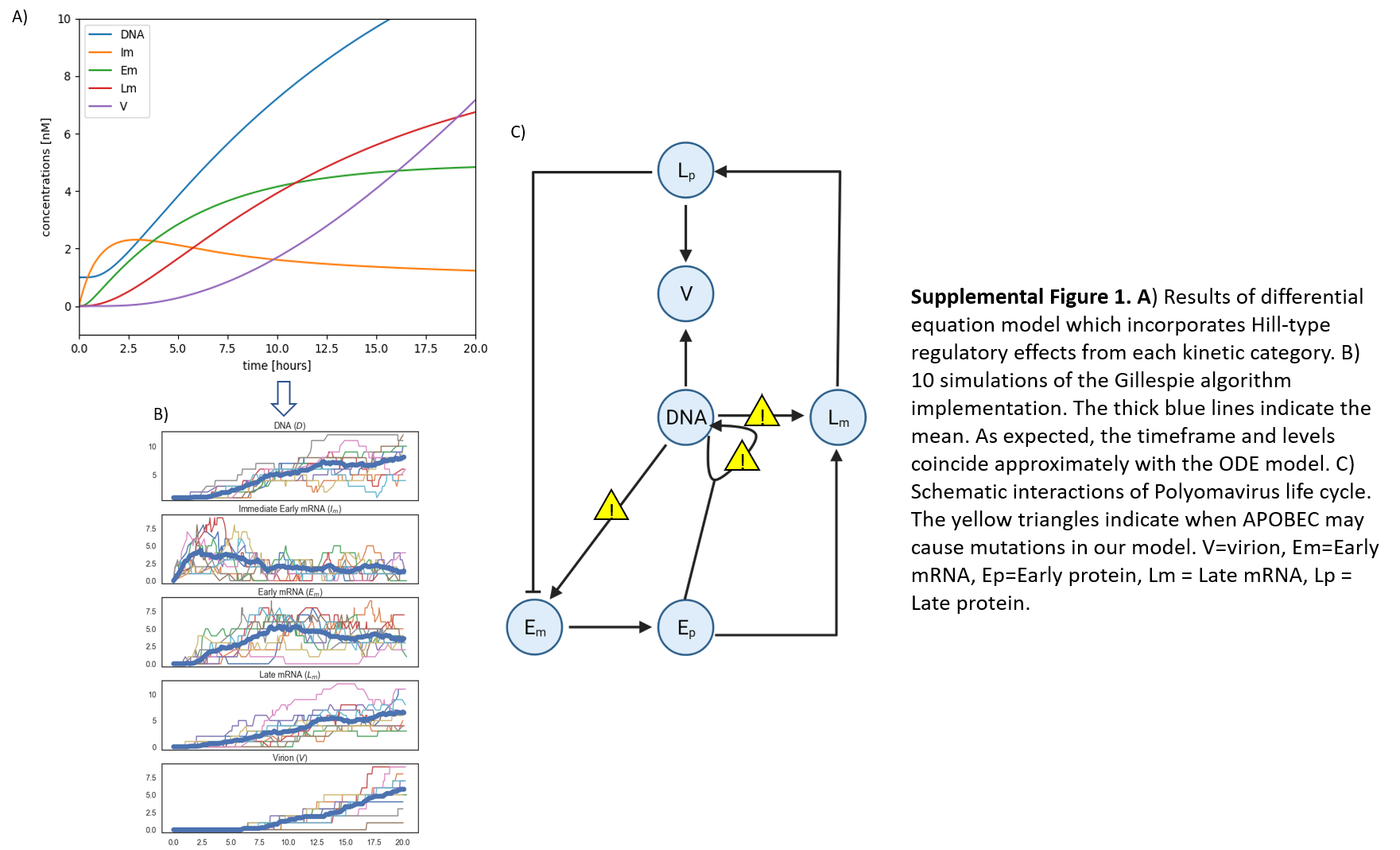

Supplement: S1 Fig — A) Results of differential equation model which incorporates Hill-type regulatory effects from each kinetic category. B) 10 simulations of the Gillespie algorithm implementation. The thick blue lines indicate the mean. As expected, the timeframe and levels coincide approximately with the ODE model. C) Schematic interactions of Polyomavirus life cycle. The yellow triangles indicate when APOBEC may cause mutations in our model. V = virion, Em = Early mRNA, Ep = Early protein, Lm = Late mRNA, Lp = Late protein. (TIF) [file ppat.1009560.s001.tif]

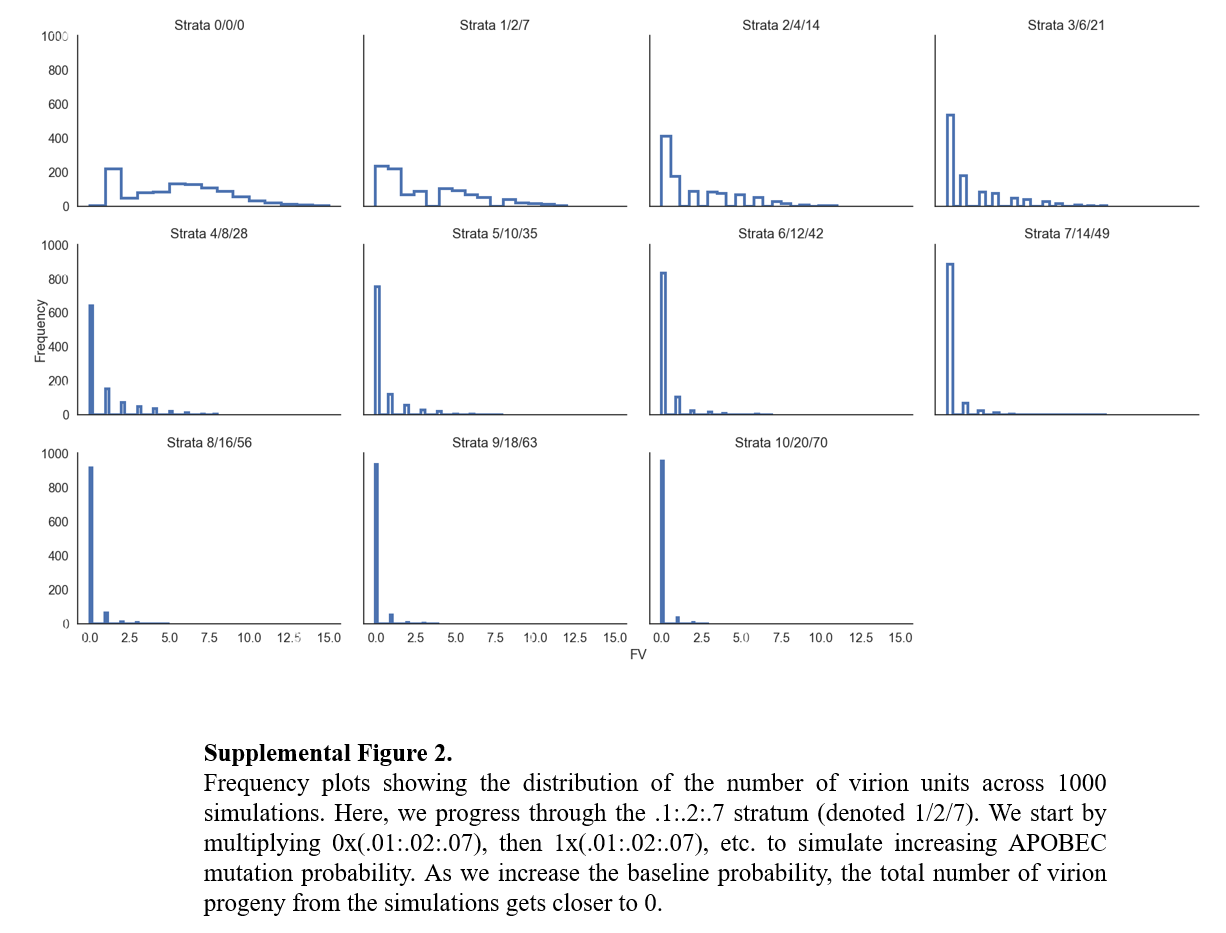

Supplement: S2 Fig — Here, we progress through the .1:.2:.7 stratum (denoted 1/2/7). We start by multiplying 0x(.01:.02:.07), then 1x(.01:.02:.07), etc. to simulate increasing APOBEC mutation probability. As we increase the baseline probability, the total number of virion progeny from the simulations gets closer to 0. (PNG) [file ppat.1009560.s002.png]

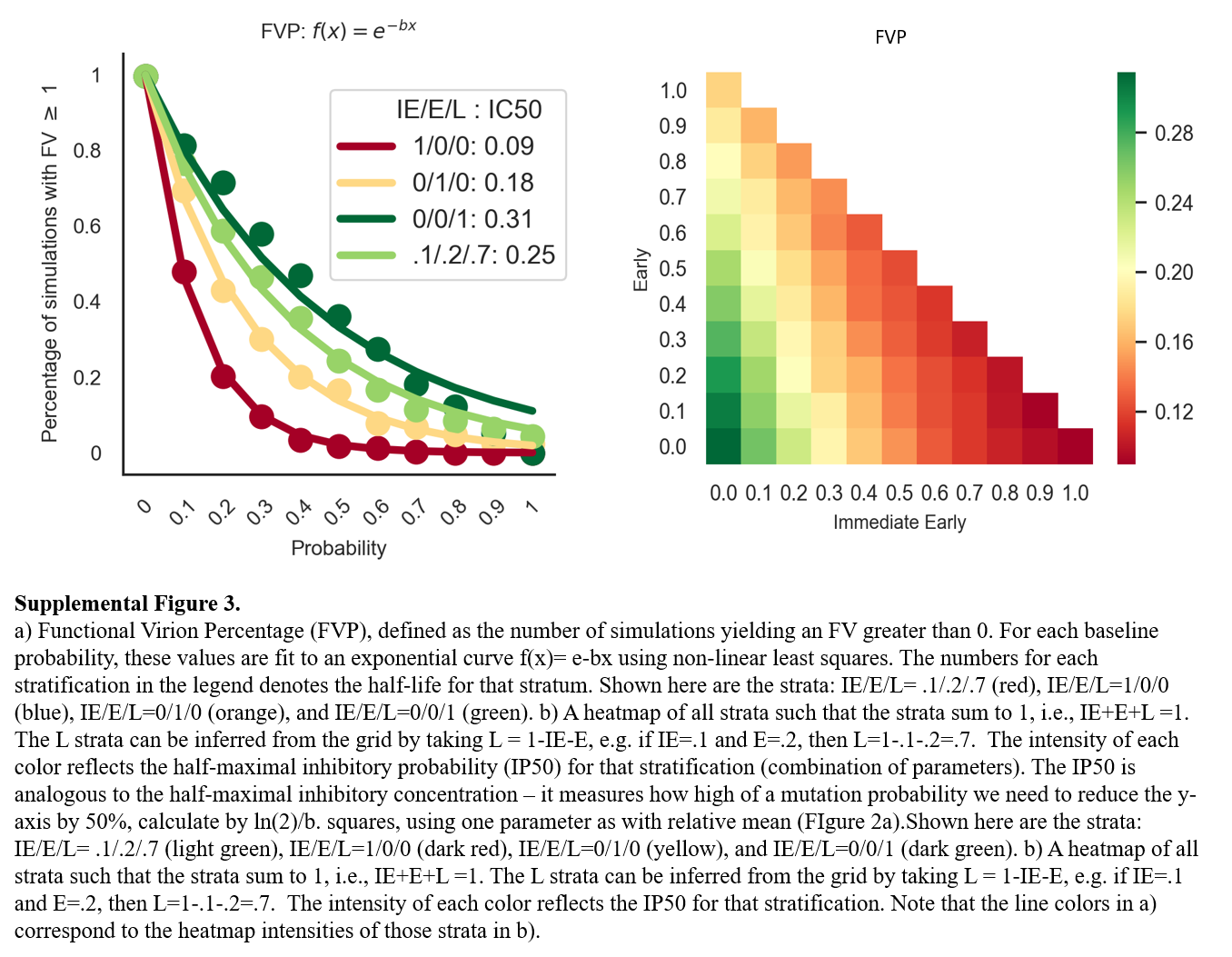

Supplement: S3 Fig — Left: Functional Virion Percentage (FVP), defined as the number of simulations yielding an FV greater than 0. For each baseline probability, these values are fit to an exponential curve f(x) = e-bx using non-linear least squares. The numbers for each stratification in the legend denotes the half-life for that stratum. Shown here are the strata: IE/E/L = .1/.2/.7 (red), IE/E/L = 1/0/0 (blue), IE/E/L = 0/1/0 (orange), and IE/E/L = 0/0/1 (green). b) A heatmap of all strata such that the strata sum to 1, i.e., IE+E+L = 1. The L strata can be inferred from the grid by taking L = 1-IE-E, e.g. if IE = .1 and E = .2, then L = 1-.1-.2 = .7. The intensity of each color reflects the half-maximal inhibitory probability (IP50) for that stratification (combination of parameters). The IP50 is analogous to the half-maximal inhibitory concentration–it measures how high of a mutation probability we need to reduce the y-axis by 50%, calculate by ln(2)/b. squares, using one parameter as with relative mean (Fig 2A). Shown here are the strata: IE/E/L = .1/.2/.7 (light green), IE/E/L = 1/0/0 (dark red), IE/E/L = 0/1/0 (yellow), and IE/E/L = 0/0/1 (dark green). Right: A heatmap of all strata such that the strata sum to 1, i.e., IE+E+L = 1. The L strata can be inferred from the grid by taking L = 1-IE-E, e.g. if IE = .1 and E = .2, then L = 1-.1-.2 = .7. The intensity of each color reflects the IP50 for that stratification. Note that the line colors in the left plot correspond to the heatmap intensities of those strata in the right plot. (PNG) [file ppat.1009560.s003.png]

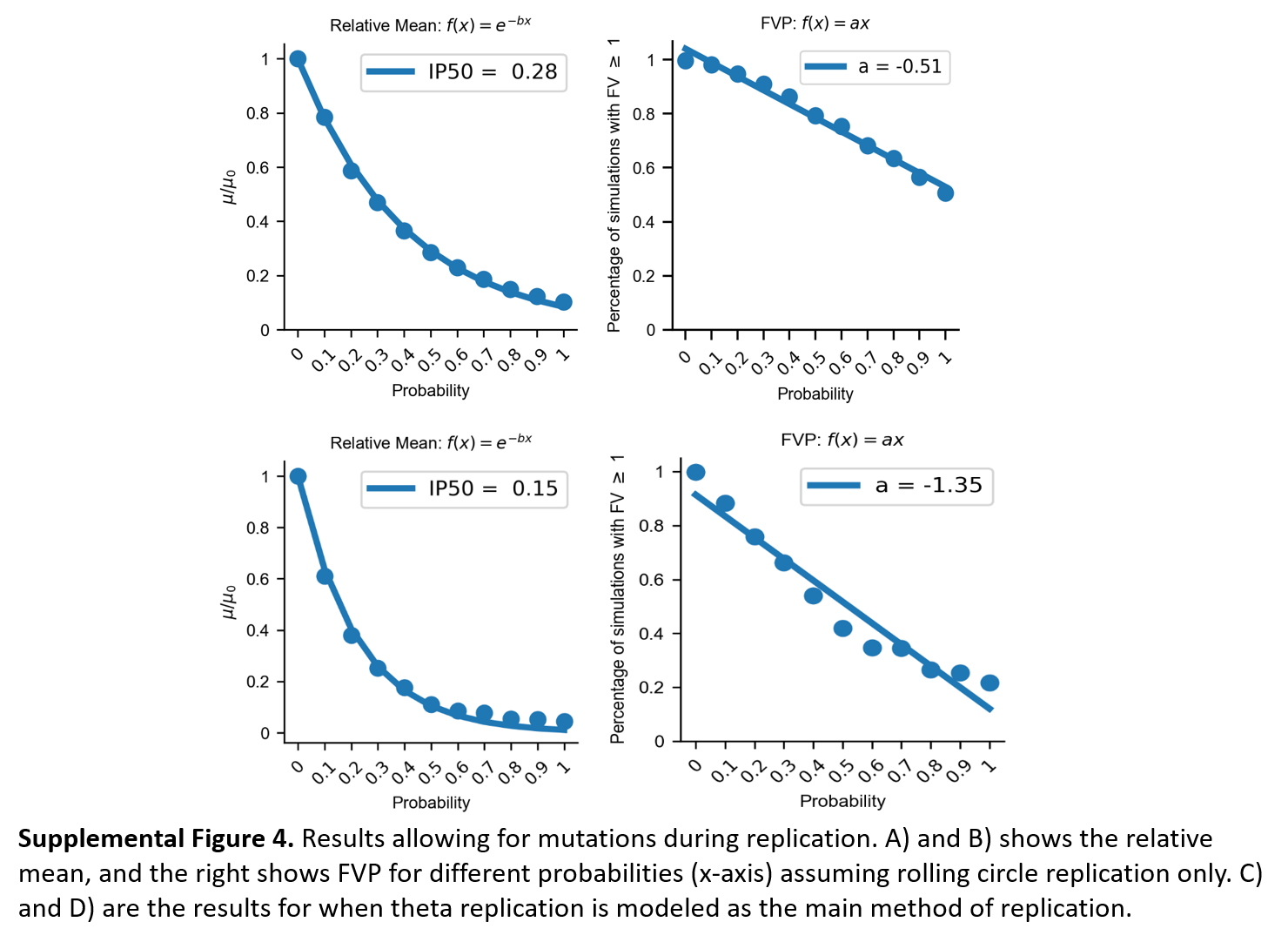

Supplement: S4 Fig — A) and B) shows the relative mean, and the right shows FVP for different probabilities (x-axis) assuming rolling circle replication only. C) and D) are the results for when theta replication is modeled as the main method of replication. (TIF) [file ppat.1009560.s004.tif]

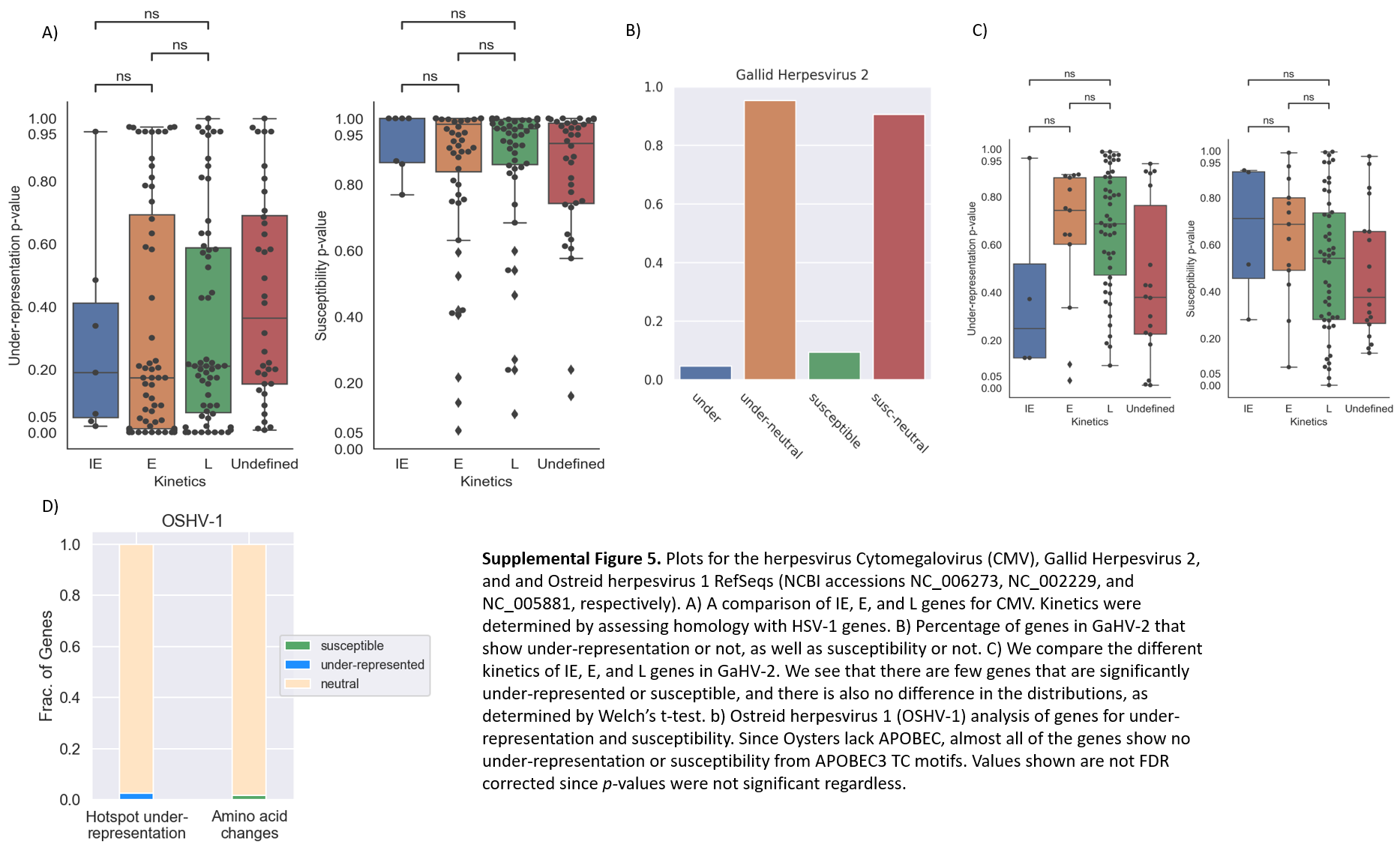

Supplement: S5 Fig — A) A comparison of IE, E, and L genes for CMV. Kinetics were determined by assessing homology with HSV-1 genes. B) Percentage of genes in GaHV-2 that show under-representation or not, as well as susceptibility or not. C) We compare the different kinetics of IE, E, and L genes in GaHV-2. We see that there are few genes that are significantly under-represented or susceptible, and there is also no difference in the distributions, as determined by Welch’s t-test. b) Ostreid herpesvirus 1 (OSHV-1) analysis of genes for under-representation and susceptibility. Since Oysters lack APOBEC, almost all of the genes show no under-representation or susceptibility from APOBEC3 TC motifs. Values shown are not FDR corrected since p-values were not significant regardless. (TIF) [file ppat.1009560.s005.tif]

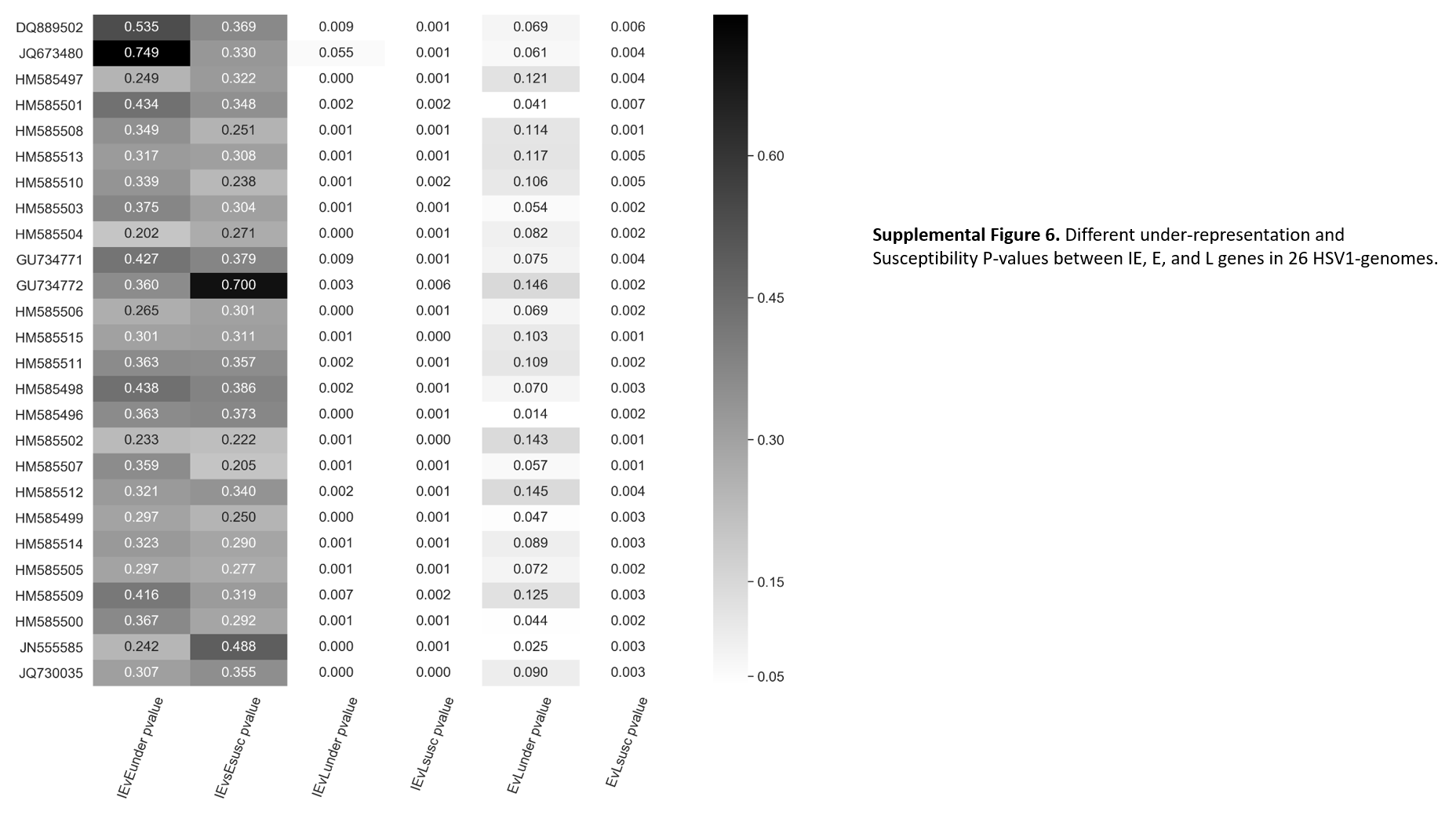

Supplement: S6 Fig — (TIF) [file ppat.1009560.s006.tif]

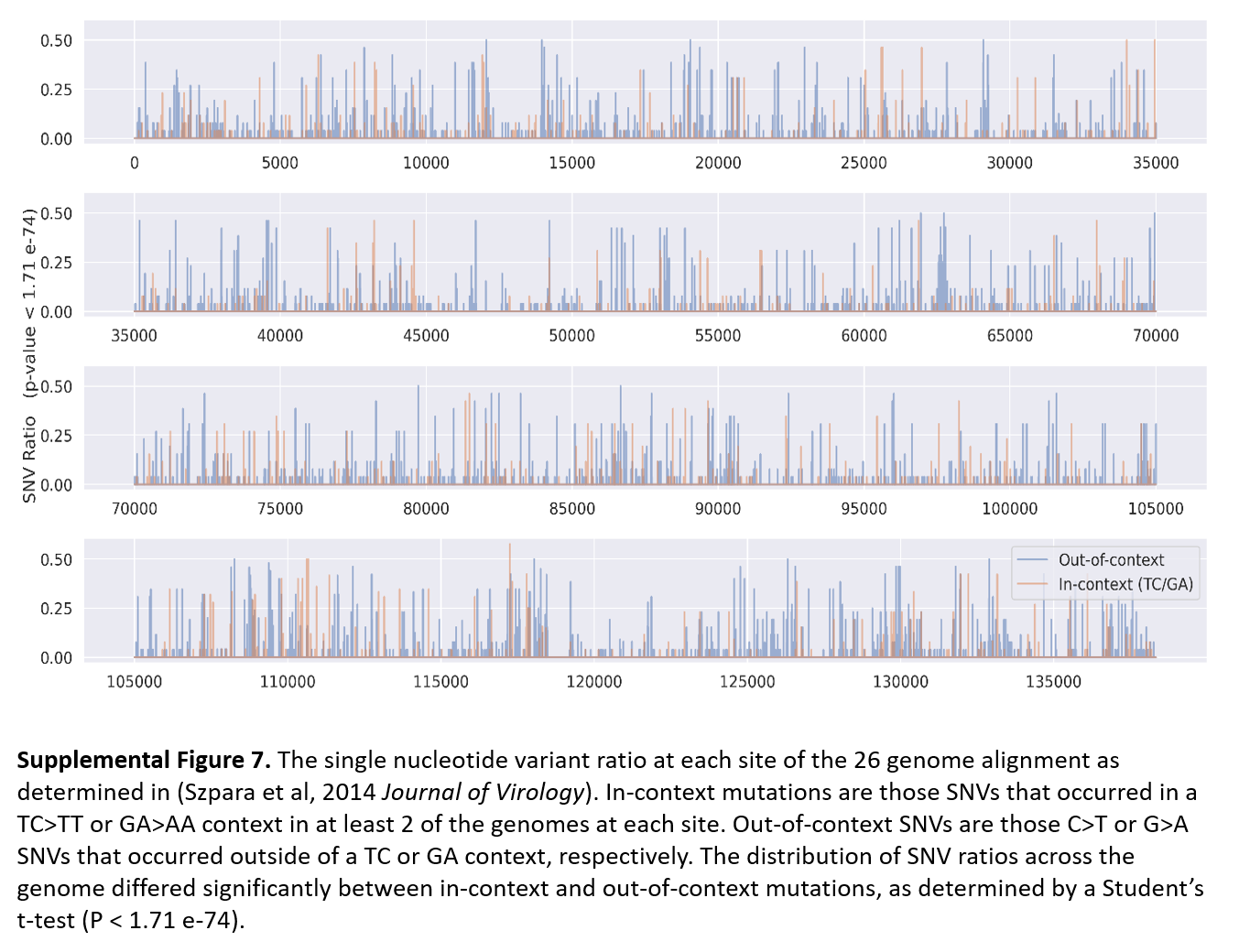

Supplement: S7 Fig — In-context mutations are those SNVs that occurred in a TC>TT or GA>AA context in at least 2 of the genomes at each site. Out-of-context SNVs are those C>T or G>A SNVs that occurred outside of a TC or GA context, respectively. The distribution of SNV ratios across the genome differed significantly between in-context and out-of-context mutations, as determined by a Student’s t-test (P < 1.71 e-74). (TIF) [file ppat.1009560.s007.tif]

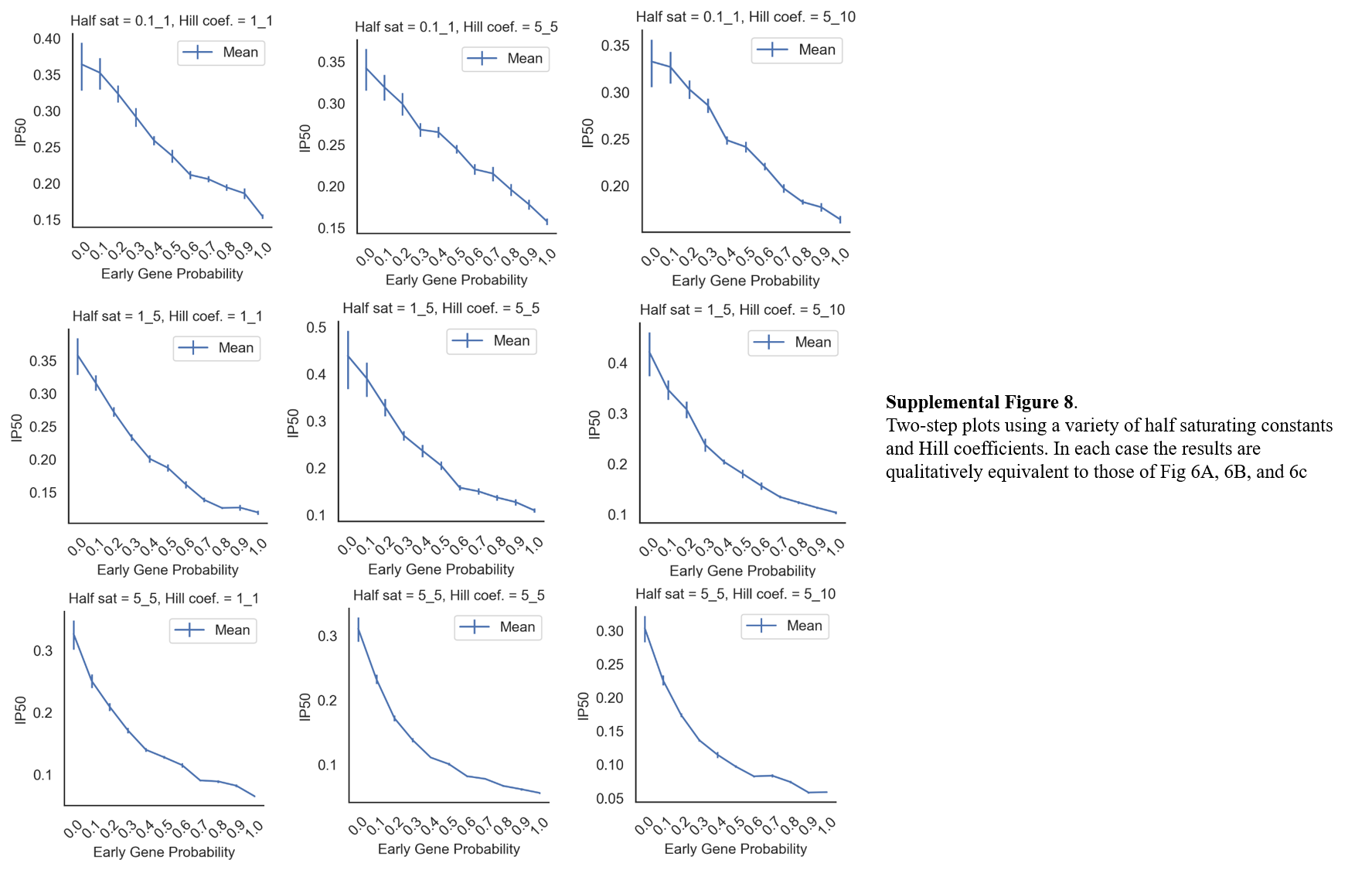

Supplement: S8 Fig — In each case the results are qualitatively equivalent to those of Fig 6A, 6B and 6C. (PNG) [file ppat.1009560.s008.png]

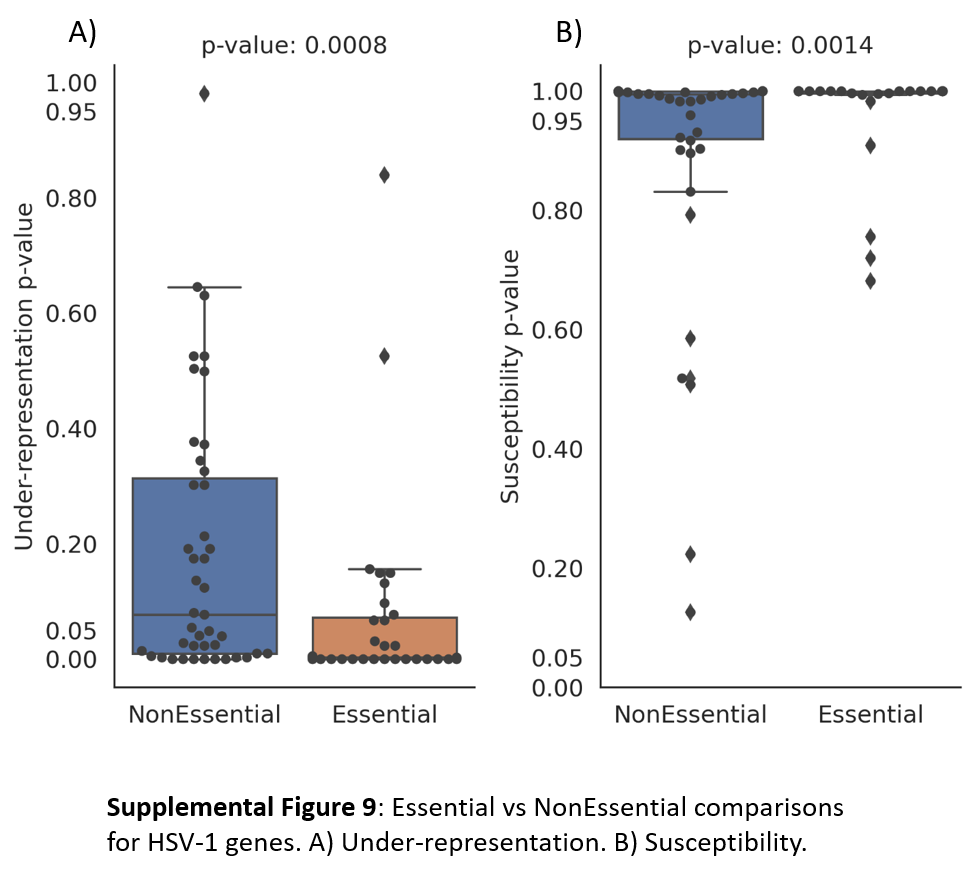

Supplement: S9 Fig — A) Under-representation. B) Susceptibility. (PNG) [file ppat.1009560.s009.png]

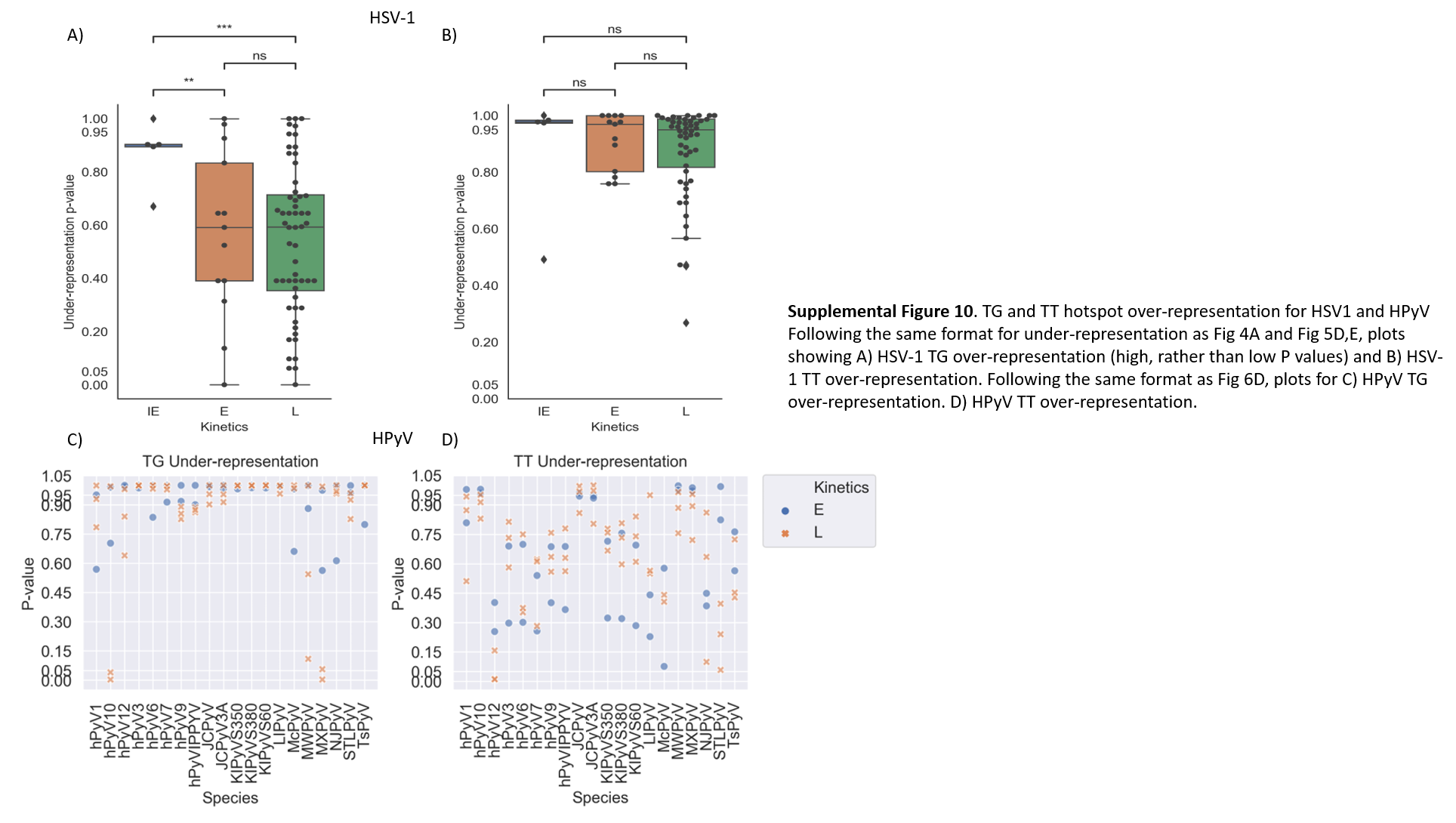

Supplement: S10 Fig — Following the same format for under-representation as Fig 4A and Fig 5D, 5E, plots showing A) HSV-1 TG over-representation (high, rather than low P values) and B) HSV-1 TT over-representation. Following the same format as Fig 6D, plots for C) HPyV TG over-representation. D) HPyV TT over-representation. (PNG) [file ppat.1009560.s010.png]
