# Supplementary material for: Mutational pressure by host APOBEC3s more strongly affects genes expressed early in the lytic phase of herpes simplex virus-1 (HSV-1) and human polyomavirus (HPyV) infection
Source: PLoS Pathog. 2021 Apr 30;17(4):e1009560. doi: 10.1371/journal.ppat.1009560 (PMC8115780; doi:10.1371/journal.ppat.1009560)
Supplement: S1 Table — (DOCX) [file ppat.1009560.s011.docx]

**Supplemental Table 1.** FVs vs mixed FV/NFV

| **Kinetics** | **Initial Functional DNA** | **Functional/Non-Functional Split** | **Combined Fisher Pval** |
| --- | --- | --- | --- |
| I | 2 | 1/1 | 1 |
| I | 10 | 5/5 | 1 |
| E | 2 | 1/1 | 1 |
| E | 10 | 5/5 | 1 |
| L | 2 | 1/1 | 1 |
| L | 10 | 5/5 | 1 |
