# Supplementary material for: Mutational pressure by host APOBEC3s more strongly affects genes expressed early in the lytic phase of herpes simplex virus-1 (HSV-1) and human polyomavirus (HPyV) infection
Source: PLoS Pathog. 2021 Apr 30;17(4):e1009560. doi: 10.1371/journal.ppat.1009560 (PMC8115780; doi:10.1371/journal.ppat.1009560)
Supplement: S2 Table — (DOCX) [file ppat.1009560.s012.docx]

**Supplemental Table 2.** CDUR analysis on 25 HSV1 genomes.

| **Accession** | **IE fraction** | **E fraction** | **L fraction** | **# CDS Analyzed** | **Under-represented** | **Susceptible** | **both** | **% under-represented** | **% susceptible** | **% both** |
| --- | --- | --- | --- | --- | --- | --- | --- | --- | --- | --- |
| DQ889502 | 3/5 | 9/13 | 26/51 | 69 | 38 | 54 | 37 | 0.550724638 | 0.782608696 | 0.536232 |
| GU734772 | 4/5 | 9/13 | 28/56 | 74 | 40 | 56 | 37 | 0.540540541 | 0.756756757 | 0.5 |
| HM585496 | 4/5 | 9/13 | 24/56 | 74 | 37 | 56 | 35 | 0.5 | 0.756756757 | 0.472973 |
| HM585497 | 4/5 | 9/13 | 27/56 | 74 | 40 | 58 | 38 | 0.540540541 | 0.783783784 | 0.513514 |
| HM585498 | 4/5 | 9/13 | 32/56 | 74 | 45 | 57 | 42 | 0.608108108 | 0.77027027 | 0.567568 |
| HM585499 | 4/5 | 9/13 | 26/56 | 74 | 39 | 58 | 38 | 0.527027027 | 0.783783784 | 0.513514 |
| HM585500 | 4/5 | 9/13 | 24/56 | 74 | 37 | 56 | 36 | 0.5 | 0.756756757 | 0.486486 |
| HM585501 | 4/5 | 9/13 | 30/56 | 74 | 43 | 58 | 40 | 0.581081081 | 0.783783784 | 0.540541 |
| HM585502 | 4/5 | 9/13 | 24/56 | 74 | 37 | 57 | 37 | 0.5 | 0.77027027 | 0.5 |
| HM585503 | 4/5 | 9/13 | 25/56 | 74 | 38 | 57 | 37 | 0.513513514 | 0.77027027 | 0.5 |
| HM585504 | 4/5 | 9/13 | 27/56 | 74 | 40 | 58 | 40 | 0.540540541 | 0.783783784 | 0.540541 |
| HM585505 | 4/5 | 9/13 | 29/56 | 74 | 42 | 57 | 39 | 0.567567568 | 0.77027027 | 0.527027 |
| HM585506 | 4/5 | 9/13 | 26/56 | 74 | 39 | 57 | 37 | 0.527027027 | 0.77027027 | 0.5 |
| HM585507 | 4/5 | 9/13 | 24/55 | 73 | 37 | 55 | 35 | 0.506849315 | 0.753424658 | 0.479452 |
| HM585508 | 4/5 | 9/13 | 29/56 | 74 | 42 | 58 | 40 | 0.567567568 | 0.783783784 | 0.540541 |
| HM585509 | 3/5 | 9/13 | 31/56 | 74 | 43 | 59 | 40 | 0.581081081 | 0.797297297 | 0.540541 |
| HM585510 | 4/5 | 9/13 | 29/56 | 74 | 42 | 60 | 40 | 0.567567568 | 0.810810811 | 0.540541 |
| HM585511 | 4/5 | 9/13 | 30/56 | 74 | 43 | 56 | 41 | 0.581081081 | 0.756756757 | 0.554054 |
| HM585512 | 4/5 | 9/13 | 26/56 | 74 | 39 | 56 | 36 | 0.527027027 | 0.756756757 | 0.486486 |
| HM585513 | 4/5 | 9/13 | 27/56 | 74 | 40 | 57 | 37 | 0.540540541 | 0.77027027 | 0.5 |
| HM585514 | 2/5 | 9/13 | 27/56 | 74 | 38 | 57 | 35 | 0.513513514 | 0.77027027 | 0.472973 |
| HM585515 | 4/5 | 9/13 | 26/56 | 74 | 39 | 55 | 36 | 0.527027027 | 0.743243243 | 0.486486 |
| JN555585 | 4/5 | 9/13 | 28/56 | 74 | 41 | 56 | 38 | 0.554054054 | 0.756756757 | 0.513514 |
| JQ673480 | 3/5 | 9/13 | 27/56 | 74 | 39 | 58 | 37 | 0.527027027 | 0.783783784 | 0.5 |
| JQ730035 | 4/5 | 9/13 | 27/56 | 74 | 40 | 55 | 36 | 0.540540541 | 0.743243243 | 0.486486 |
