# Supplementary material for: Mutational pressure by host APOBEC3s more strongly affects genes expressed early in the lytic phase of herpes simplex virus-1 (HSV-1) and human polyomavirus (HPyV) infection
Source: PLoS Pathog. 2021 Apr 30;17(4):e1009560. doi: 10.1371/journal.ppat.1009560 (PMC8115780; doi:10.1371/journal.ppat.1009560)
Supplement: S4 Table — (DOCX) [file ppat.1009560.s014.docx]

**Supplemental Table 4.** Propensities for HSV-1 Gillespie Algorithm

| **Reactions** | **Description** |
| --- | --- |
| $\boldsymbol{\alpha}_{\boldsymbol{1}}* \frac{\boldsymbol{E}^{\boldsymbol{n}_{\boldsymbol{Ep}}}}{\boldsymbol{k}_{\boldsymbol{Ep}}^{\boldsymbol{n}_{\boldsymbol{E}_{\boldsymbol{p}}}}+\boldsymbol{E}_{\boldsymbol{p}}^{\boldsymbol{n}_{\boldsymbol{E}_{\boldsymbol{p}}}}}$ | Viral DNA replication |
| $\boldsymbol{\alpha}_{\boldsymbol{2}}* \boldsymbol{D}*\boldsymbol{L}_{\boldsymbol{p}}$ | Virion creation |
| $\boldsymbol{\delta}_{\boldsymbol{D}}*\boldsymbol{D}$ | Viral DNA degradation |
| $\boldsymbol{\delta}_{\boldsymbol{V}}*\boldsymbol{V}$ | Virion degradation |
| $\boldsymbol{\gamma}_{\boldsymbol{I}_{\boldsymbol{m}}}*\left( \frac{\boldsymbol{k}_{\boldsymbol{I}_{\boldsymbol{p}}}^{\boldsymbol{n}_{\boldsymbol{I}_{\boldsymbol{p}_{\boldsymbol{I}}}}}}{\boldsymbol{k}_{\boldsymbol{I}_{\boldsymbol{p}}}^{\boldsymbol{n}_{\boldsymbol{I}_{\boldsymbol{p}_{\boldsymbol{I}}}}}+\boldsymbol{I}_{\boldsymbol{p}}^{\boldsymbol{n}_{\boldsymbol{I}_{\boldsymbol{p}_{\boldsymbol{I}}}}}}- \frac{\boldsymbol{L}_{\boldsymbol{p}}^{\boldsymbol{n}_{\boldsymbol{L}_{\boldsymbol{p}_{\boldsymbol{I}}}}}}{\boldsymbol{k}_{\boldsymbol{L}_{\boldsymbol{p}}}^{\boldsymbol{n}_{\boldsymbol{L}_{\boldsymbol{p}_{\boldsymbol{I}}}}}+\boldsymbol{L}_{\boldsymbol{p}}^{\boldsymbol{n}_{\boldsymbol{L}_{\boldsymbol{p}_{\boldsymbol{I}}}}}} \right)$ | IE transcription |
| $\boldsymbol{\delta}_{\boldsymbol{I}_{\boldsymbol{m}}}*\boldsymbol{I}_{\boldsymbol{m}}$ | IE mRNA degradation |
| $\boldsymbol{\beta}_{\boldsymbol{I}_{\boldsymbol{p}}}*\boldsymbol{I}_{\boldsymbol{m}}$ | IE mRNA translation |
| $\boldsymbol{\delta}_{\boldsymbol{I}_{\boldsymbol{P}}}*\boldsymbol{I}_{\boldsymbol{P}}$ | IE protein degradation |
| $\boldsymbol{\gamma}_{\boldsymbol{E}_{\boldsymbol{m}}}* \frac{\boldsymbol{I}_{\boldsymbol{p}}^{\boldsymbol{n}_{\boldsymbol{I}_{\boldsymbol{p}_{\boldsymbol{E}}}}}}{\boldsymbol{I}_{\boldsymbol{p}}^{\boldsymbol{n}_{\boldsymbol{I}_{\boldsymbol{p}_{\boldsymbol{E}}}}}+\boldsymbol{k}_{\boldsymbol{E}_{\boldsymbol{p}}}^{\boldsymbol{n}_{\boldsymbol{I}_{\boldsymbol{p}_{\boldsymbol{E}}}}}}$ | E mRNA transcription |
| $\boldsymbol{\delta}_{\boldsymbol{E}_{\boldsymbol{m}}}*\boldsymbol{E}_{\boldsymbol{m}}$ | E mRNA degradation |
| $\boldsymbol{\beta}_{\boldsymbol{E}_{\boldsymbol{p}}}*\boldsymbol{E}_{\boldsymbol{m}}$ | E mRNA translation |
| $\boldsymbol{\delta}_{\boldsymbol{E}_{\boldsymbol{p}}}*\boldsymbol{E}_{\boldsymbol{p}}$ | E protein degradation |
| $\boldsymbol{\gamma}_{\boldsymbol{L}_{\boldsymbol{m}}}* \frac{\boldsymbol{D}^{\boldsymbol{n}_{\boldsymbol{D}}}}{\boldsymbol{k}_{\boldsymbol{D}}^{\boldsymbol{n}_{\boldsymbol{D}}}+\boldsymbol{D}^{\boldsymbol{n}_{\boldsymbol{D}}}}* \frac{\boldsymbol{I}_{\boldsymbol{p}}^{\boldsymbol{n}_{\boldsymbol{I}_{\boldsymbol{p}_{\boldsymbol{L}}}}}}{\boldsymbol{k}_{\boldsymbol{L}_{\boldsymbol{p}}}^{\boldsymbol{n}_{\boldsymbol{I}_{\boldsymbol{p}_{\boldsymbol{L}}}}}+\boldsymbol{I}_{\boldsymbol{p}}^{\boldsymbol{n}_{\boldsymbol{I}_{\boldsymbol{p}_{\boldsymbol{L}}}}}}$ | L mRNA transcription |
| $\boldsymbol{\delta}_{\boldsymbol{L}_{\boldsymbol{m}}}*\boldsymbol{L}_{\boldsymbol{m}}$ | L mRNA degradation |
| $\boldsymbol{\beta}_{\boldsymbol{L}_{\boldsymbol{p}}}*\boldsymbol{L}_{\boldsymbol{m}}$ | L mRNA translation |
| $\boldsymbol{\delta}_{\boldsymbol{L}_{\boldsymbol{p}}}*\boldsymbol{L}_{\boldsymbol{p}}$ | L protein degradation |
