# Supplementary material for: Mutational pressure by host APOBEC3s more strongly affects genes expressed early in the lytic phase of herpes simplex virus-1 (HSV-1) and human polyomavirus (HPyV) infection
Source: PLoS Pathog. 2021 Apr 30;17(4):e1009560. doi: 10.1371/journal.ppat.1009560 (PMC8115780; doi:10.1371/journal.ppat.1009560)
Supplement: S5 Table — (DOCX) [file ppat.1009560.s015.docx]

**Supplemental Table 5.** Propensities for two-step Gillespie Model

| **Reactions** | **Description** |
| --- | --- |
| $\boldsymbol{\alpha}_{\boldsymbol{1}}\boldsymbol{*}\frac{\boldsymbol{E}^{\boldsymbol{n}_{\boldsymbol{Ep}}}}{\boldsymbol{k}_{\boldsymbol{Ep}}^{\boldsymbol{n}_{\boldsymbol{E}_{\boldsymbol{p}}}}\boldsymbol{+}\boldsymbol{E}_{\boldsymbol{p}}^{\boldsymbol{n}_{\boldsymbol{E}_{\boldsymbol{p}}}}}$ | Viral DNA replication |
| $\boldsymbol{\alpha}_{\boldsymbol{2}}\boldsymbol{* D*}\boldsymbol{L}_{\boldsymbol{p}}$ | Virion creation |
| $\boldsymbol{\delta}_{\boldsymbol{D}}\boldsymbol{*D}$ | DNA degradation |
| $\boldsymbol{\delta}_{\boldsymbol{V}}\boldsymbol{*V}$ | Virion degradation |
| $\boldsymbol{\gamma}_{\boldsymbol{E}_{\boldsymbol{m}}}\boldsymbol{*}\left( \frac{\boldsymbol{k}_{\boldsymbol{E}_{\boldsymbol{p}}}^{\boldsymbol{n}_{\boldsymbol{E}_{\boldsymbol{p}_{\boldsymbol{E}}}}}}{\boldsymbol{k}_{\boldsymbol{E}_{\boldsymbol{p}}}^{\boldsymbol{n}_{\boldsymbol{E}_{\boldsymbol{p}_{\boldsymbol{E}}}}}\boldsymbol{+}\boldsymbol{E}_{\boldsymbol{p}}^{\boldsymbol{n}_{\boldsymbol{E}_{\boldsymbol{p}_{\boldsymbol{E}}}}}}\boldsymbol{-}\frac{\boldsymbol{L}_{\boldsymbol{p}}^{\boldsymbol{n}_{\boldsymbol{L}_{\boldsymbol{p}_{\boldsymbol{E}}}}}}{\boldsymbol{k}_{\boldsymbol{L}_{\boldsymbol{p}}}^{\boldsymbol{n}_{\boldsymbol{L}_{\boldsymbol{p}_{\boldsymbol{E}}}}}\boldsymbol{+}\boldsymbol{L}_{\boldsymbol{p}}^{\boldsymbol{n}_{\boldsymbol{L}_{\boldsymbol{E}}}}} \right)$ | E mRNA transcription |
| $\boldsymbol{\delta}_{\boldsymbol{E}_{\boldsymbol{m}}}\boldsymbol{*}\boldsymbol{E}_{\boldsymbol{m}}$ | E mRNA degradation |
| $\boldsymbol{\beta}_{\boldsymbol{E}_{\boldsymbol{p}}}\boldsymbol{*}\boldsymbol{E}_{\boldsymbol{m}}$ | E mRNA translation |
| $\boldsymbol{\delta}_{\boldsymbol{E}_{\boldsymbol{p}}}\boldsymbol{*}\boldsymbol{E}_{\boldsymbol{p}}$ | E protein degradation |
| $\boldsymbol{\gamma}_{\boldsymbol{L}_{\boldsymbol{m}}}\boldsymbol{*}\frac{\boldsymbol{D}^{\boldsymbol{n}_{\boldsymbol{D}}}}{\boldsymbol{k}_{\boldsymbol{D}}^{\boldsymbol{n}_{\boldsymbol{D}}}\boldsymbol{+}\boldsymbol{D}^{\boldsymbol{n}_{\boldsymbol{D}}}}\boldsymbol{*}\frac{\boldsymbol{E}_{\boldsymbol{p}}^{\boldsymbol{n}_{\boldsymbol{E}_{\boldsymbol{p}_{\boldsymbol{L}}}}}}{\boldsymbol{k}_{\boldsymbol{L}_{\boldsymbol{p}}}^{\boldsymbol{n}_{\boldsymbol{E}_{\boldsymbol{p}_{\boldsymbol{L}}}}}\boldsymbol{+}\boldsymbol{E}_{\boldsymbol{p}}^{\boldsymbol{n}_{\boldsymbol{E}_{\boldsymbol{p}_{\boldsymbol{L}}}}}}$ | L mRNA transcription |
| $\boldsymbol{\delta}_{\boldsymbol{L}_{\boldsymbol{m}}}\boldsymbol{*}\boldsymbol{L}_{\boldsymbol{m}}$ | L mRNA degradation |
| $\boldsymbol{\beta}_{\boldsymbol{L}_{\boldsymbol{p}}}\boldsymbol{*}\boldsymbol{L}_{\boldsymbol{m}}$ | L mRNA translation |
| $\boldsymbol{\delta}_{\boldsymbol{L}_{\boldsymbol{p}}}\boldsymbol{*}\boldsymbol{L}_{\boldsymbol{p}}$ | L protein degradation |
