# Supplementary material for: Mutational pressure by host APOBEC3s more strongly affects genes expressed early in the lytic phase of herpes simplex virus-1 (HSV-1) and human polyomavirus (HPyV) infection
Source: PLoS Pathog. 2021 Apr 30;17(4):e1009560. doi: 10.1371/journal.ppat.1009560 (PMC8115780; doi:10.1371/journal.ppat.1009560)
Supplement: S6 Table — (DOCX) [file ppat.1009560.s016.docx]

**Supplemental Table 6.** Units in equations

| **Parameter** | **Units** |
| --- | --- |
| $\boldsymbol{\alpha}_{\boldsymbol{1}}$ | nMh^-1^ |
| $\boldsymbol{\alpha}_{\boldsymbol{2}}$ | nM^-1^h^-1^ |
| $\boldsymbol{\delta}$**^*^** | h^-1^ |
| $\boldsymbol{\gamma}_{\boldsymbol{I}_{\boldsymbol{m}}}$ | nMh^-1^ |
| $\boldsymbol{\gamma}_{\boldsymbol{E}_{\boldsymbol{m}}}$ | nMh^-1^ |
| $\boldsymbol{\gamma}_{\boldsymbol{L}_{\boldsymbol{m}}}$ | nMh^-1^ |
| $\boldsymbol{\beta}_{\boldsymbol{I}_{\boldsymbol{p}}}$ | h^-1^ |
| $\boldsymbol{\beta}_{\boldsymbol{E}_{\boldsymbol{p}}}$ | h^-1^ |
| $\boldsymbol{\beta}_{\boldsymbol{L}_{\boldsymbol{p}}}$ | h^-1^ |

*Units for all decay parameters
